# Structure of bis­(tetra­ethyl­ammonium) molybdate dihydrate obtained by the reaction between ammonium molybdate and tetra­ethyl­ammonium hydroxide

**DOI:** 10.1107/S2056989026008078

**Published:** 2026-08-11

**Authors:** P. A. S. N. P. Jayawardena, Teodore J. Melnyczuk-Gould, A. M. Buddhika Chandima, Stanislav Groysman, Cassandra L. Ward

**Affiliations:** a Department of Chemistry Wayne State University, 5101 Cass Avenue Detroit Michigan 48202 USA; b Lumigen Instrument Center Wayne State University, 5101 Cass Avenue Detroit Michigan 48202 USA; Texas A & M University, USA

**Keywords:** tetra­ethyl­ammonium molybdate, crystal structure, hydrate, hydrogen bonding

## Abstract

Treatment of ammonium molybdate with tetra­ethyl­ammonium hydroxide produces tetra­ethyl­ammonium molybdate that can be recrystallized from aceto­nitrile/ether. The crystal structure reveals the dihydrate compound (C_8_H_20_N)_2_[MoO_4_]·2H_2_O. The presence of water mol­ecules in the structure is further supported by FTIR, which demonstrates the corresponding broad signal centered around 3225 cm^−1^. The supra­molecular structure of (Et_4_N)_2_[MoO_4_]·2H_2_O demonstrates two-dimensional layers of tetra­hedral molybdate mol­ecules bridged by water mol­ecules, with each Mo-oxo serving as a hydrogen-bond acceptor. The molybdate/water layers are spaced by the layers of disordered tetra­ethyl­ammonium counter-ions. The structural parameters of (Et_4_N)_2_[MoO_4_]·2H_2_O are presented at 100 and 200 K.

## Abstract 2

The crystal structure of tetra­ethyl­ammonium molybdate dihydrate reveals two-dimensional layers of tetra­hedral molybdate mol­ecules bridged by water mol­ecules, with each Mo-oxo serving as a hydrogen bond acceptor.

## 1. Chemical context

**Figure 1:**
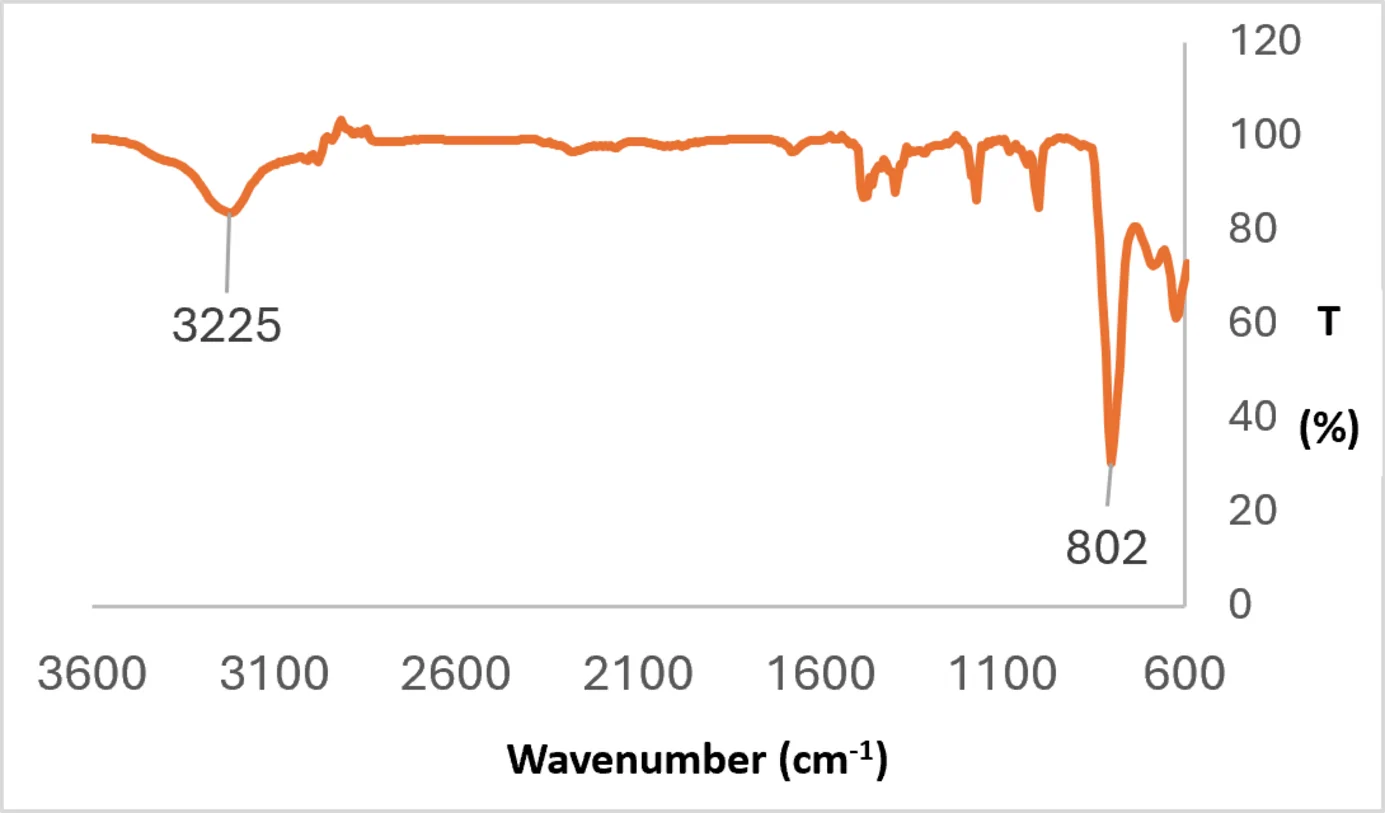
FTIR spectrum of (Et_4_N)_2_[MoO_4_]·2H_2_O.

Tetra­ethyl­ammonium molybdate (Et_4_N)_2_[MoO_4_] is a common precursor in the chemistry of high-oxidation state molyb­denum(VI). It contains a nucleophilic molybdenum-oxo functionality, which undergoes reactions with a variety of electrophiles, including protonation by catechols or benzene­dithiols (Groysman *et al.*, 2008 ▸; Chandima *et al.*, 2026 ▸; Kaluarachchige Don *et al.*, 2021 ▸, 2023*a* ▸,*b* ▸, 2024 ▸), silylation and alkyl­ation with silicon/carbon electrophiles (Partyka & Holm, 2004 ▸; Song *et al.*, 2020 ▸), or direct reaction with coordinatively unsaturated/electrophilic metal centers (Yan *et al.*, 2013 ▸; Chandima *et al.*, 2025 ▸; Cotton *et al.*, 2001 ▸; Cisse *et al.*, 2003 ▸). Tetra­ethyl­ammonium molybdate can be prepared by the reaction of ammonium molybdate (NH_4_)_2_[MoO_4_] with two equivalents of tetra­ethyl­ammonium hydroxide (Cotton *et al.*, 2001 ▸), or by a salt metathesis of silver molybdate Ag_2_MoO_4_ with tetra­ethyl­ammonium chloride (Knopf *et al.*, 2014 ▸). However, its crystal structure has not been previously reported. Herein we describe the structure of (Et_4_N)_2_[MoO_4_] hydrate measured at two different temperatures, along with its spectroscopic characterization data.
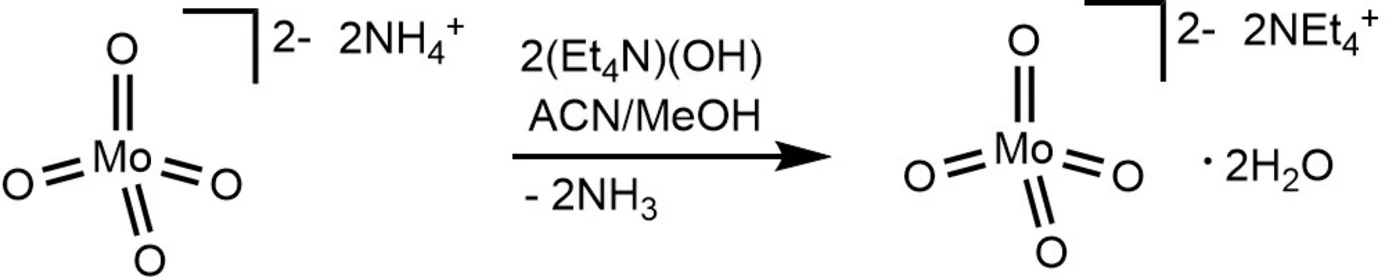


(Et_4_N)_2_[MoO_4_] was obtained by the treatment of (NH_4_)_2_[MoO_4_] with two equivalents of tetra­ethyl­ammonium hydroxide (see below for the full synthetic details). The crude solid was dried for 24 h at 333 K. The crude product can be recrystallized from aceto­nitrile/ether by vapor diffusion, giving a recrystallization yield of 64%. Recrystallization of crude tetra­ethyl­ammonium molybdate from aceto­nitrile/ether under dry conditions produces colorless blocks of (Et_4_N)_2_[MoO_4_]·2H_2_O, which were characterized by X-ray crystallography (see below), IR, NMR, and UV-vis spectroscopy. The IR spectrum (Fig. 1 ▸) shows a strong and broad signal at 802 cm^−1^ (consistent with multiple Mo=O stretches), and a very broad signal around 3225 cm^−1^ (consistent with O—H stretches).

## 2. Structural commentary

**Figure 2:**
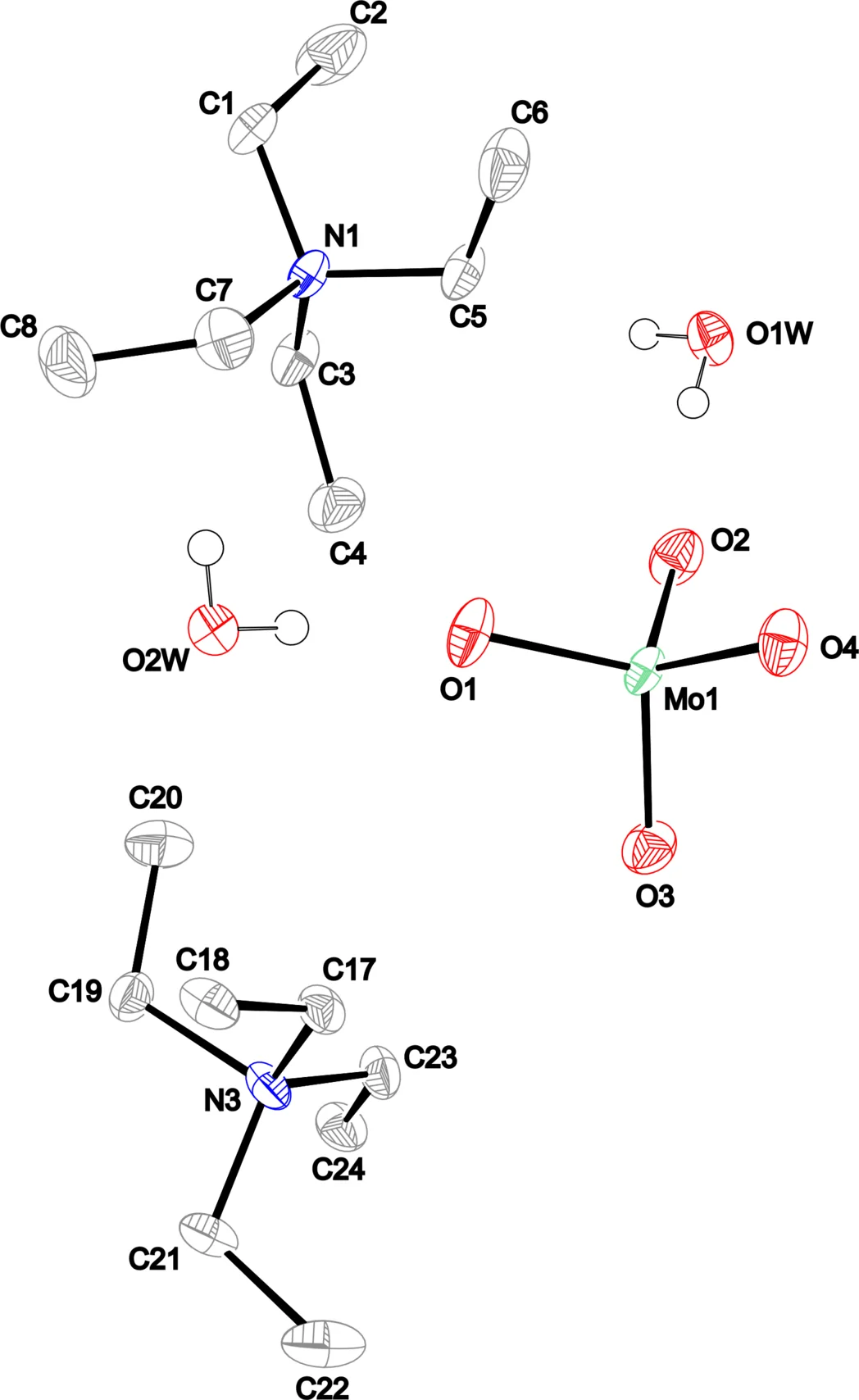
The structure of (Et_4_N)_2_[MoO_4_]·2H_2_O with 50% probability ellipsoids. The alternative conformation of one of the tetra­ethyl­ammonium ions is not shown.

The structure of (Et_4_N)_2_[MoO_4_]·2H_2_O is shown in Fig. 2 ▸. The compound crystallizes in the *P*-1 space group at 100 K. The asymmetric unit contains one molybdate anion, two water mol­ecules, one fully occupied tetra­ethyl­ammonium ion (Et_4_N^+^), and two half-occupied Et_4_N^+^ cations located on inversion centers. Difference-Fourier maps indicate that the disordered cations adopt the all-*trans (tt·tt*) conformer. The alternative *trans–gauche* (*tg·tg*) conformer was evaluated for both inversion-disordered Et_4_N^+^ cations; however, this model gave an *R* factor greater than 8%, and the corresponding difference-Fourier maps were inconsistent with the modeled structure. The Mo^VI^ center adopts a nearly ideal tetra­hedral geometry (Table 1 ▸), with Mo–oxo bond distances comparable to those reported for related group 6 structures (Khranenko *et al.*, 2018 ▸; Partyka & Holm, 2004 ▸).

## 3. Supra­molecular features

**Figure 3:**
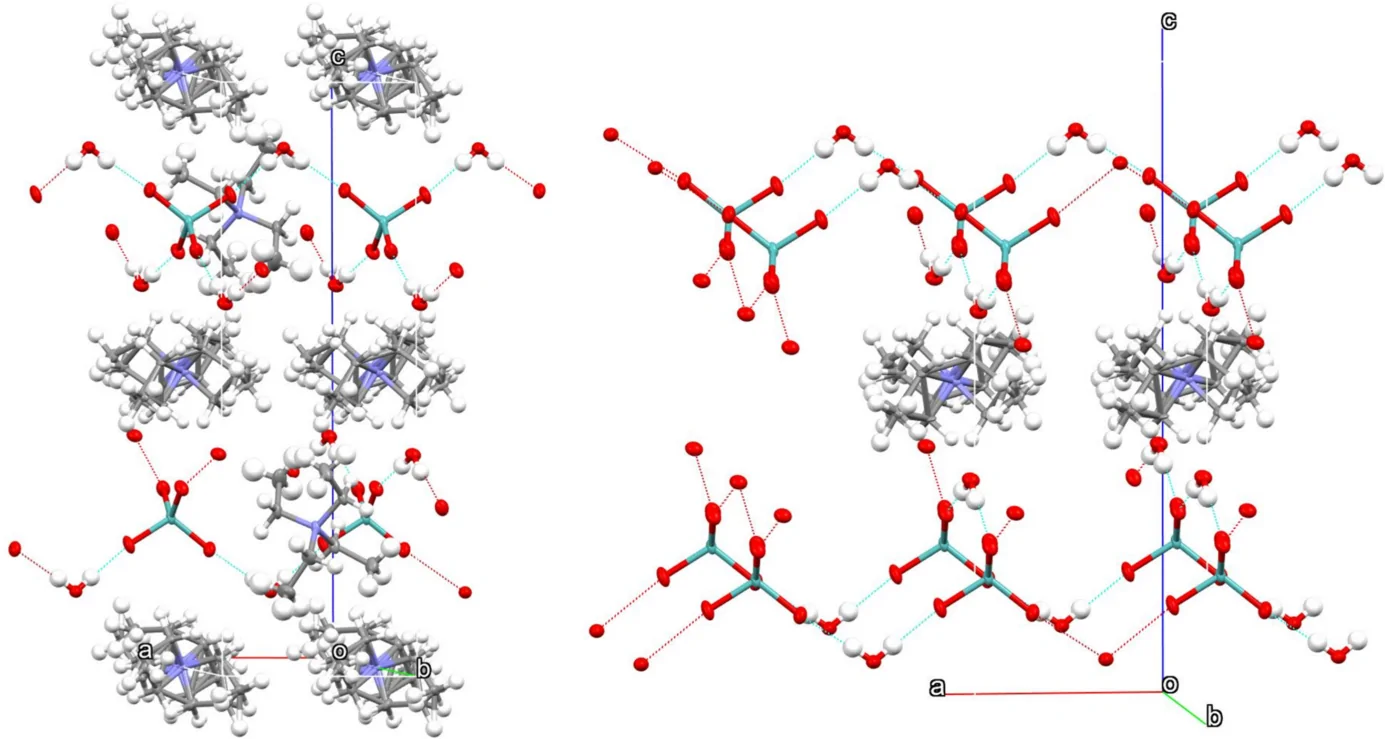
Supra­molecular structure of (Et_4_N)_2_[MoO_4_]·2H_2_O showing (left) a single unit-cell packing and (right) the layers of molybdate–water spaced by the layers of disordered tetra­ethyl­ammonium ions. Teal = Mo atoms; red = O atoms; lavender = N atoms; white = H atoms.

An important aspect of the structure of (Et_4_N)_2_[MoO_4_]·2H_2_O is the presence of water mol­ecules that bridge individual [MoO_4_]^2−^ centers [Mo=O⋯H—OH = 1.94 (2) Å]. Each Mo-oxo functionality serves as the hydrogen-bond acceptor, with the water mol­ecule linking it to four neighboring [MoO_4_]^2−^ centers located in the same plane (Table 2 ▸, Fig. 3 ▸). This bonding pattern results in the formation of dense two-dimensional parallel sheets that also include the ordered tetra­ethyl­ammonium ions. In contrast, the disordered tetra­ethyl­ammonium cations form buffer layers separating the adjacent [MoO_4_]^2−^/H_2_O sheets.

## 4. Temperature dependence study

**Figure 4:**
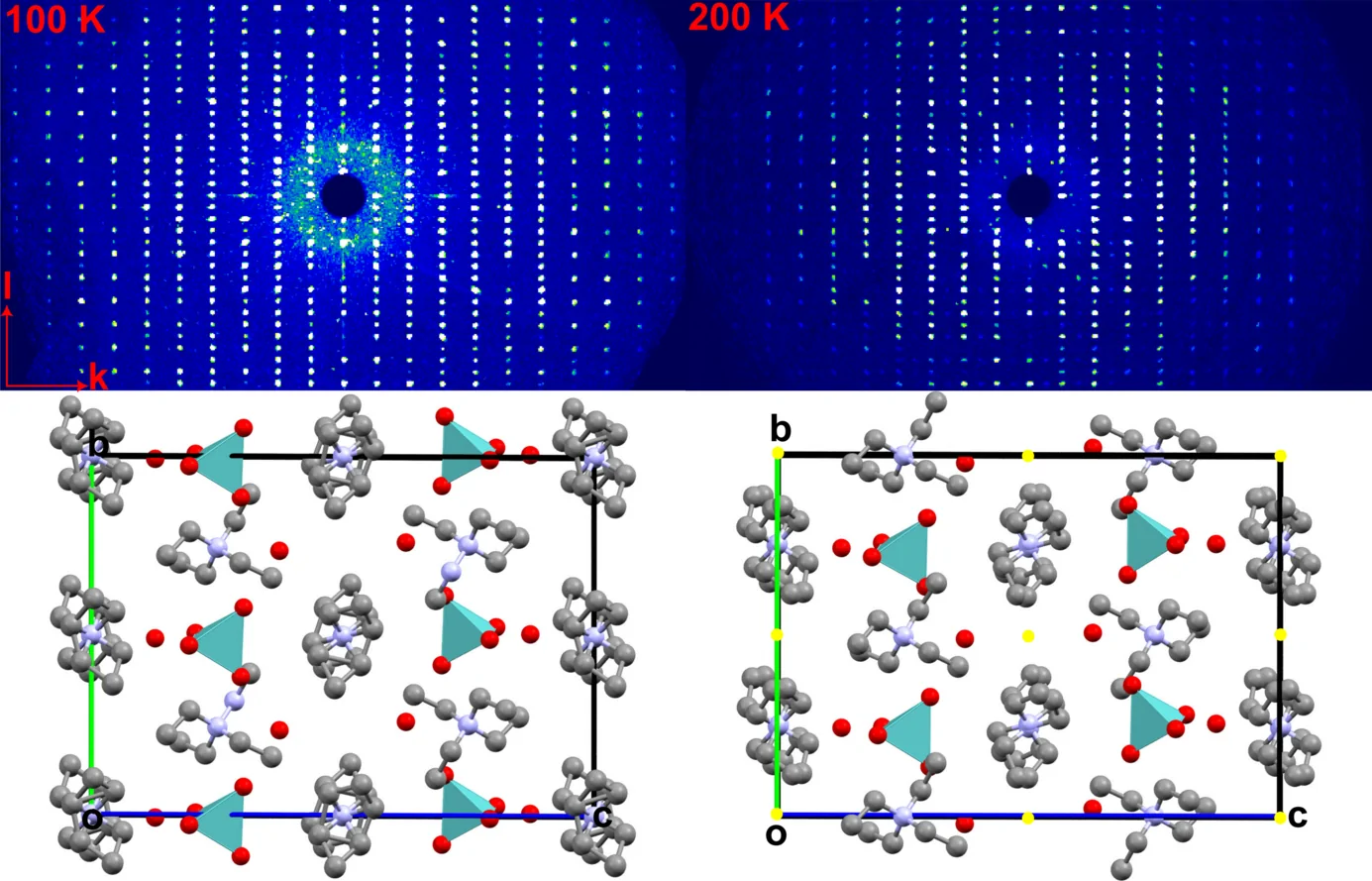
Comparison of synthesized precession images of the 0*kl* plane collected at 100 K and 200 K showing the additional weak reflections along *k*. The corresponding unit-cell packing diagrams viewed along [100] are shown below for the 100 K structure (right) and the 200 K structure (left). For comparison, the 100 K cell is shown doubled along the *b* axis to illustrate that the disorder is retained, as observed in the smaller unit cell. Yellow spheres in the 200 K packing diagram indicate inversion centers. The disordered Et_4_N^+^ cations are positioned on these inversion centers and are therefore not visible in the packing view. Teal = Mo atoms; red = O atoms; lavender = N atoms; white = H atoms.

The crystal was initially mounted directly at 100 K, and diffraction data were collected at this temperature. Upon warming to 200 K, additional weak reflections were observed (Fig. 4 ▸, top) corresponding to a doubling of the *b* axis. The structure was still solved in *P*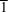, but the corresponding increase in the unit-cell volume doubled *Z* to 4. This elongation of the *b* axis appears to arise from a reduction in the symmetry-related disorder of the Et_4_N^+^ cations, which shift the cations away from the inversion centers to general positions, which requires expansion of the unit cell (Fig. 4 ▸). At 200 K, the Et_4_N^+^ ions remain disordered, but the occupancies refined to 94:6 and 88:12 for the two disordered cations. Both cations were also modeled as the *tt·tt* conformer. However, after re-cooling the crystal to 100 K, the original unit cell was not recovered, indicating that the transition is irreversible. Warming likely allows the Et_4_N^+^ ions to adopt a more thermodynamically favorable arrangement within the lattice; therefore, slow cooling may favor formation of the larger unit cell, whereas rapid cooling directly to 100 K traps the cations in the smaller, disordered, unit cell. Data at 100 K were collected using longer integration times to ensure that a supercell was not overlooked. In addition, solving the structure with the *b* axis doubled did not remove the disorder imposed by symmetry (Fig. 4 ▸). Although the unit-cell metrics are nearly monoclinic, attempts to index the 200 K data in a higher-symmetry monoclinic cell were poor, with an *R*_sym_ value of 0.5. However, a monoclinic polymorph may exist at higher temperatures.

Further temperature-dependent unit-cell determinations were performed with an Oxford Cryo-system 800 at a cooling rate of 360 K h^−1^. A crystal was mounted at 293 K under the nitro­gen stream. Inter­estingly, the 100 K unit cell was recovered, and the structure was solved (*R* = 4.7%). However, weak reflections along the *c* axis were noticed and a structure solution was attempted by doubling the *c* axis. Structure solution of the doubled-*c* cell gave poor structure metrics (*R* = 15.5%), indicating that the apparent superstructure was either incorrect or complicated by another crystallographic issue. Cooling this crystal to 100 K at 360 K h^−1^ led to indexing of the 200 K cell. A second crystal mounted directly at 100 K reproduced the original unit cell, and upon warming to 200 K (360 K h^−1^), showed the same unit-cell transition. However, upon warming to 250 K, the 100 K cell was recovered, but the weak additional reflections emerged along the *c* axis and they became more intense at 265 K. Data were collected at 265 K where structure refinement in the doubled-*c* cell was slightly poorer quality than the 100 K unit cell (*R* = 8% *vs R* = 6%, respectively). The reciprocal lattice also showed weak satellite peaks along the *a* axis indicating potential modulation. It is unclear if the crystal is degrading, fracturing, or becoming modulated above 250 K, leading to the additional observed weak reflections. Overall, these temperature-dependent changes point to lattice instability and reorganization of the Et_4_N^+^ cations and the 100 K-to-200 K unit-cell change may correspond to an alternative metastable phase where ordering of the cations occurs.

## 5. Database survey

A survey of the Cambridge Structural Database Web (CSD, accessed July 2026; Groom *et al.*, 2016 ▸) indicates that no structures of tetra­ethyl­ammonium molybdate or tungstate salts have been previously reported. A related compound, (Et_4_N)_2_[WO_3_S], CSD refcode 265169, was reported by Partyka & Holm (2004 ▸); however, no hydrogen bonding is observed in this structure. Several mol­ecular tungstate species containing [WO_4_]^2−^ with other counter-ions, such as [Ni(1,2-di­amino­ethane)_3_]^2+^, are also present in the database (refcode 1831059; Khranenko *et al.*, 2018 ▸). These structures exhibit overall similar tetra­hedral geometries at the M^VI^ metal center. Matsumoto and co-workers reported Na_2_MoO_4_·2H_2_O, which displays alternating layers of tetra­hedral MoO_4_^2−^ anions and water mol­ecules, with inter­linking sodium ions and a hydrogen-bonding network similar to that observed in the present structure (refcode 1592854; Matsumoto *et al.*, 1975 ▸). Román and co-workers reported (C_4_H_12_N)_2_[MoO_4_], which contains two molybdate anions and four *tert*-butyl­ammonium cations in the asymmetric unit (refcode 1174192; Román *et al.*, 1994 ▸). Although no water mol­ecules are present in this structure, the crystal packing is consolidated by an extensive network of hydrogen contacts between the O atoms of MoO_4_^2−^ and the N atom of the cations.

Several salts containing Et_4_N^+^ cations have been reported to exhibit crystallographic disorder. For example, (Et_4_N)_2_[NiCl_4_] and (Et_4_N)_2_[CoCl_4_] crystallize in tetra­gonal unit cells at 295 K, with the Et_4_N^+^ cations disordered by symmetry (refcode 1243784; Stucky *et al.*, 1967 ▸). Phase transitions have also been described for molybdate-containing salts. For example, [NiEn_3_]MoO_4_ undergoes a reversible phase transition at 300 K, accompanied by a change in space group (refcode 1876331; Sukhikh *et al.*, 2019 ▸). In another example, crystals of (Me_4_N)_2_[MnBr_4_] transform from a monoclinic to an ortho­rhom­bic unit cell at 276 K, concurrent with reduced cation disorder (refcode 2513934; Shin *et al.*, 2026 ▸). Similarly, (Et_4_N)[ReS_4_] undergoes a phase transition in which the Et_4_N^+^ cation is highly disordered at room temperature but becomes ordered at 285 K (refcode 1974401; Bernhardt & Herbst-Irmer, 2020 ▸). Notably, (Et_4_N)[ReS_4_] also forms a metastable phase upon rapid cooling to 110 K. Twinning has been reported in many structures that undergo related phase transitions, arising from either merohedral or reticular twinning (refcode 1974401, Bernhardt & Herbst-Irmer, 2020 ▸; 1876331, Sukhikh *et al.*, 2019 ▸; 996406–996408, Lutz *et al.*, 2014 ▸). However, twinning does not appear to be significant in the present structures at any of the temperatures examined.

## 6. Synthesis and crystallization

The outline of this procedure was previously reported (Cotton *et al.*, 2001 ▸). Ammonium molybdate, (NH_4_)_2_[MoO_4_] (2.00 g, 10.2 mmol, 1 equiv.), was dissolved in anhydrous aceto­nitrile (500.0 mL), and the suspension was purged with Ar for 30 min. Tetra­ethyl­ammonium hydroxide (NEt_4_)(OH) (25 wt% in methanol, *ca*. 1.5 *M*, 13.7 mL, 20.4 mmol, 2.0 equiv.) was measured under an Ar atmosphere and added dropwise to the reaction mixture. The solution was further purged with argon for 30 min and then stirred overnight at ambient temperature. After completion of the reaction, any remaining insoluble material was removed by filtration, and the filtrate was concentrated under reduced pressure. The resulting residue was redissolved in aceto­nitrile. If incomplete dissolution (<90%) was observed, the solution was filtered, concentrated, and redissolved in aceto­nitrile; this process was repeated until most of the material was soluble. Diethyl ether was then added dropwise until cloudiness persisted, affording precipitation of the product. The solid was isolated and dried under vacuum for 24 h at 333–343 K. The crude product of (Et_4_N)_2_[MoO_4_]·2H_2_O (1.60 g, 3.51 mmol, 34%) was then transferred to the glove box. This solid (100 mg) can be recrystallized from aceto­nitrile/diethyl ether, forming colorless plates (64 mg, 0.14 mmol, 64% recrystallization yield). ^1^H NMR (CD_3_CN, 400 MHz) δ 3.27 (*q*, *J* = 8.0 Hz, 8H, NC*H*_2_CH_3_), 1.24 (*m*, 12H, NCH_2_C*H*_3_) ppm. IR (ATR) 3220 (*br*, OH), 1486 (*m*), 1386 (*m*), 1173 (*m*), 1002 (*m*), 802 (*vs*, Mo=O), 702 (*m*), 633 (*m*) cm^−1^. UV-vis (CH_3_CN) ∼240 nm (LMCT).

## 7. Refinement

Crystal data, data collection and structure refinement details are summarized in Table 3 ▸. The hydrogen atoms of the water mol­ecules were located from difference-Fourier (Δ*F*) maps, whereas all remaining hydrogen atoms were placed in calculated positions and refined isotropically using a riding model.

## Supplementary Material

Crystal structure: contains datablock(s) 100k, 200k. DOI: 10.1107/S2056989026008078/jy2075sup1.cif

Structure factors: contains datablock(s) 100k. DOI: 10.1107/S2056989026008078/jy2075100ksup2.hkl

Structure factors: contains datablock(s) 200k. DOI: 10.1107/S2056989026008078/jy2075200ksup3.hkl

CCDC references: 2579006, 2579005

Additional supporting information:  crystallographic information; 3D view; checkCIF report

## Figures and Tables

**Table 1:** Selected geometric parameters (Å, °) at 100 K[Chem scheme1]

Mo1—O1	1.7636 (9)	Mo1—O3	1.7593 (10)
Mo1—O2	1.7654 (10)	Mo1—O4	1.7668 (9)
			
O1—Mo1—O2	109.45 (5)	O3—Mo1—O1	109.36 (5)
O1—Mo1—O4	109.38 (5)	O3—Mo1—O2	109.50 (5)
O2—Mo1—O4	109.27 (5)	O3—Mo1—O4	109.86 (5)

**Table 2:** Hydrogen-bond geometry (Å, °) at 100 K[Chem scheme1]

*D*—H⋯*A*	*D*—H	H⋯*A*	*D*⋯*A*	*D*—H⋯*A*
O1*W*—H1*WA*⋯O2	0.85 (2)	1.93 (2)	2.7693 (14)	173 (2)
O1*W*—H1*WB*⋯O4^i^	0.82 (2)	1.93 (2)	2.7466 (14)	174 (2)
O2*W*—H2*WA*⋯O1	0.83 (2)	1.93 (2)	2.7539 (15)	177 (2)
O2*W*—H2*WB*⋯O3^ii^	0.80 (2)	1.95 (2)	2.7462 (15)	177 (2)
C1—H1*A*⋯O3^iii^	0.99	2.45	3.3193 (17)	146
C1—H1*B*⋯O1*W*^ii^	0.99	2.52	3.4147 (17)	149
C3—H3*A*⋯O2*W*^i^	0.99	2.46	3.4043 (17)	158
C3—H3*B*⋯O4^i^	0.99	2.39	3.3081 (17)	154
C4—H4*B*⋯O2	0.98	2.51	3.4881 (18)	173
C5—H5*A*⋯O1*W*	0.99	2.59	3.4059 (17)	140
C7—H7*B*⋯O2^ii^	0.99	2.59	3.5499 (18)	163
C9—H9*A*⋯O4	0.99	2.29	3.057 (3)	134
C12—H12*B*⋯O1*W*^iv^	0.98	2.50	3.159 (5)	124
C13—H13*B*⋯O1*W*	0.99	2.51	3.461 (3)	162
C15—H15*B*⋯O4^iv^	0.99	2.48	3.432 (3)	162
C21—H21*B*⋯O3^v^	0.99	2.46	3.168 (3)	128

Symmetry codes: (i) 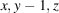; (ii) 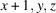; (iii) 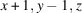; (iv) 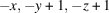; (v) 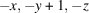.

**Table 3:** Experimental details

	100 K	200 K
Crystal data		
Chemical formula	(C_8_H_20_N)_2_[MoO_4_]·2H_2_O	(C_8_H_20_N)_2_[MoO_4_]·2H_2_O
*M*_r_	456.47	456.47
Crystal system, space group	Triclinic, *P* 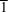	Triclinic, *P* 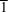
Temperature (K)	100	200
*a*, *b*, *c* (Å)	7.3202 (2), 7.3753 (2), 20.6328 (8)	7.4299 (2), 14.7269 (4), 20.6213 (7)
α, β, γ (°)	96.227 (1), 90.233 (1), 90.746 (1)	90.065 (1), 95.821 (1), 90.898 (1)
*V* (Å^3^)	1107.25 (6)	2244.45 (11)
*Z*	2	4
Radiation type	Mo *K*α	Mo *K*α
μ (mm^−1^)	0.62	0.61
Crystal size (mm)	0.15 × 0.15 × 0.01	0.15 × 0.15 × 0.01

Data collection		
Diffractometer	Bruker D8 VENTURE	Bruker D8 VENTURE
Absorption correction	Multi-scan (*SADABS*; Krause *et al.*, 2015 ▸)	Multi-scan (*SADABS*; Krause *et al.*, 2015 ▸)
*T*_min_, *T*_max_	0.712, 0.746	0.710, 0.746
No. of measured, independent and observed [*I* > 2σ(*I*)] reflections	114550, 6451, 6035	95151, 10335, 8514
*R*_int_	0.066	0.055
(sin θ/λ)_max_ (Å^−1^)	0.704	0.650

Refinement		
*R*[*F*^2^ > 2σ(*F*^2^)], *wR*(*F*^2^), *S*	0.021, 0.051, 1.11	0.028, 0.064, 1.04
No. of reflections	6451	10335
No. of parameters	325	659
No. of restraints	262	912
H-atom treatment	H atoms treated by a mixture of independent and constrained refinement	H atoms treated by a mixture of independent and constrained refinement
Δρ_max_, Δρ_min_ (e Å^−3^)	0.37, −0.44	0.33, −0.46

Computer programs: *APEX6* and *SAINT* (Bruker, 2016 ▸), *SHELXT2018/2* (Sheldrick, 2015*a* ▸), *SHELXL2025/1* (Sheldrick, 2015*b* ▸) and *OLEX2* (Dolomanov *et al.*, 2009 ▸).

## supplementary crystallographic information

### Bis(tetraethylammonium) molybdate dihydrate (100k) Crystal data

**Table:**

(C_8_H_20_N)_2_[MoO_4_]·2H_2_O	*Z* = 2
*M**_r_* = 456.47	*F*(000) = 488
Triclinic, *P*1	*D*_x_ = 1.369 Mg m^−^^3^
*a* = 7.3202 (2) Å	Mo *K*α radiation, λ = 0.71073 Å
*b* = 7.3753 (2) Å	Cell parameters from 9759 reflections
*c* = 20.6328 (8) Å	θ = 3.0–30.0°
α = 96.227 (1)°	µ = 0.62 mm^−^^1^
β = 90.233 (1)°	*T* = 100 K
γ = 90.746 (1)°	Plate, colourless
*V* = 1107.25 (6) Å^3^	0.15 × 0.15 × 0.01 mm

### Bis(tetraethylammonium) molybdate dihydrate (100k) Data collection

**Table:**

Bruker D8 VENTURE diffractometer	6451 independent reflections
Radiation source: microfocus sealed tube, Incoatec IµS	6035 reflections with *I* > 2σ(*I*)
Multilayer mirror monochromator	*R*_int_ = 0.066
φ and ω scans	θ_max_ = 30.0°, θ_min_ = 2.9°
Absorption correction: multi-scan (SADABS; Krause *et al.*, 2015)	*h* = −10→10
*T*_min_ = 0.712, *T*_max_ = 0.746	*k* = −10→10
114550 measured reflections	*l* = −29→29

### Bis(tetraethylammonium) molybdate dihydrate (100k) Refinement

**Table:**

Refinement on *F*^2^	Primary atom site location: dual
Least-squares matrix: full	Hydrogen site location: mixed
*R*[*F*^2^ > 2σ(*F*^2^)] = 0.021	H atoms treated by a mixture of independent and constrained refinement
*wR*(*F*^2^) = 0.051	*w* = 1/[σ^2^(*F*_o_^2^) + (0.0154*P*)^2^ + 0.5961*P*] where *P* = (*F*_o_^2^ + 2*F*_c_^2^)/3
*S* = 1.11	(Δ/σ)_max_ = 0.001
6451 reflections	Δρ_max_ = 0.37 e Å^−^^3^
325 parameters	Δρ_min_ = −0.44 e Å^−^^3^
262 restraints	

### Bis(tetraethylammonium) molybdate dihydrate (100k) Special details

**Table:**

Geometry. All esds (except the esd in the dihedral angle between two l.s. planes) are estimated using the full covariance matrix. The cell esds are taken into account individually in the estimation of esds in distances, angles and torsion angles; correlations between esds in cell parameters are only used when they are defined by crystal symmetry. An approximate (isotropic) treatment of cell esds is used for estimating esds involving l.s. planes.
Refinement. A suitable colorless crystal was mounted on a MicroMount (MiTeGen) using Paratone oil (Parabar 10312, Hampton Research) and placed on a Bruker D8 Venture diffractometer equipped with kappa geometry, an Incoatec IµS microfocus X-ray source producing Mo *K*α radiation, and a multilayer mirror monochromator. Diffraction intensities were measured with a Photon III CPAD area detector positioned 40 mm from the crystal. Data were collected at 100 K using an Oxford Cryosystems 800 Cryostream low-temperature apparatus. Data reduction and integration were performed with *SAINT* V8.40b in *APEX6* v2025.6-0, and a multiscan absorption correction was applied using *SADABS-2016/2*. The structure was solved by the dual-space method implemented in *SHELXT* (Sheldrick, 2015a) and refined by full-matrix least-squares methods against *F*^2^ using *SHELXL-2025/1* (Sheldrick, 2015*b*) within *Olex2* (Dolomanov *et al.*, 2009). All non-hydrogen atoms were refined anisotropically. Used SIMU and SADI restraints for the disordered TEA cations. Used DFIX constraint for the O-H on the water molecules. OMITTED reflections due to beam stop.

### Bis(tetraethylammonium) molybdate dihydrate (100k) Fractional atomic coordinates and isotropic or equivalent isotropic displacement parameters (Å^2^)

**Table:**

	*x*	*y*	*z*	*U*_iso_*/*U*_eq_	Occ. (<1)
Mo1	0.03026 (2)	0.46031 (2)	0.24950 (2)	0.01650 (3)	
O1	0.23054 (13)	0.42370 (14)	0.20295 (5)	0.0286 (2)	
O2	−0.01266 (14)	0.27015 (13)	0.29257 (5)	0.0280 (2)	
O3	−0.15511 (13)	0.48933 (14)	0.19725 (5)	0.0269 (2)	
O4	0.05987 (14)	0.65724 (13)	0.30561 (5)	0.0267 (2)	
O1W	0.01580 (15)	−0.00465 (14)	0.37334 (5)	0.0263 (2)	
H1WA	0.012 (3)	0.074 (2)	0.3464 (9)	0.039*	
H1WB	0.032 (3)	−0.102 (2)	0.3510 (9)	0.039*	
O2W	0.52735 (15)	0.47877 (15)	0.12552 (5)	0.0269 (2)	
H2WA	0.440 (2)	0.458 (3)	0.1490 (9)	0.040*	
H2WB	0.618 (2)	0.481 (3)	0.1476 (9)	0.040*	
N1	0.50831 (13)	−0.02227 (14)	0.24919 (5)	0.0178 (2)	
C1	0.65532 (18)	−0.14098 (19)	0.27344 (7)	0.0249 (3)	
H1A	0.705169	−0.218814	0.235686	0.030*	
H1B	0.756067	−0.061268	0.292475	0.030*	
C2	0.5923 (3)	−0.2618 (3)	0.32360 (10)	0.0457 (5)	
H2A	0.495319	−0.344490	0.304877	0.068*	
H2B	0.545354	−0.186282	0.361752	0.068*	
H2C	0.695321	−0.333333	0.336791	0.068*	
C3	0.36154 (17)	−0.14608 (18)	0.21585 (7)	0.0246 (3)	
H3A	0.419892	−0.230638	0.181768	0.029*	
H3B	0.308486	−0.220631	0.248375	0.029*	
C4	0.2084 (2)	−0.0513 (2)	0.18483 (8)	0.0347 (3)	
H4A	0.257659	0.017358	0.150588	0.052*	
H4B	0.148587	0.032699	0.217981	0.052*	
H4C	0.119130	−0.142148	0.165728	0.052*	
C5	0.42116 (17)	0.09769 (18)	0.30451 (7)	0.0242 (3)	
H5A	0.346185	0.019643	0.330450	0.029*	
H5B	0.337794	0.183048	0.285714	0.029*	
C6	0.5547 (2)	0.2075 (2)	0.34987 (9)	0.0362 (4)	
H6A	0.633217	0.281962	0.324578	0.054*	
H6B	0.630260	0.124460	0.372170	0.054*	
H6C	0.487107	0.287134	0.382212	0.054*	
C7	0.5938 (2)	0.1010 (2)	0.20346 (8)	0.0301 (3)	
H7A	0.499955	0.186615	0.191125	0.036*	
H7B	0.692031	0.174429	0.227250	0.036*	
C8	0.6733 (2)	0.0042 (3)	0.14190 (8)	0.0452 (4)	
H8A	0.576333	−0.064480	0.116728	0.068*	
H8B	0.767772	−0.079966	0.153316	0.068*	
H8C	0.727294	0.094090	0.115732	0.068*	
N2	0.000000	0.500000	0.500000	0.0351 (5)	
C9	0.0995 (4)	0.5136 (4)	0.43841 (12)	0.0237 (5)	0.5
H9A	0.017432	0.564169	0.406825	0.028*	0.5
H9B	0.135312	0.390057	0.419586	0.028*	0.5
C10	0.2700 (5)	0.6347 (6)	0.4488 (2)	0.0313 (8)	0.5
H10A	0.315814	0.663672	0.406537	0.047*	0.5
H10B	0.364220	0.570314	0.470848	0.047*	0.5
H10C	0.239442	0.747794	0.475767	0.047*	0.5
C11	−0.0578 (4)	0.6459 (3)	0.54040 (13)	0.0223 (5)	0.5
H11A	0.049199	0.721358	0.556504	0.027*	0.5
H11B	−0.119376	0.602271	0.578548	0.027*	0.5
C12	−0.1905 (7)	0.7636 (7)	0.5057 (2)	0.0290 (9)	0.5
H12A	−0.135054	0.795596	0.465224	0.043*	0.5
H12B	−0.216522	0.875312	0.534197	0.043*	0.5
H12C	−0.304466	0.695101	0.495526	0.043*	0.5
C13	−0.1571 (3)	0.3494 (4)	0.47810 (12)	0.0205 (5)	0.5
H13A	−0.235304	0.397190	0.444752	0.025*	0.5
H13B	−0.097667	0.238329	0.457233	0.025*	0.5
C14	−0.2781 (5)	0.2958 (5)	0.53232 (19)	0.0281 (7)	0.5
H14A	−0.373062	0.210102	0.513985	0.042*	0.5
H14B	−0.335396	0.404882	0.554329	0.042*	0.5
H14C	−0.204264	0.237701	0.563767	0.042*	0.5
C15	0.1298 (3)	0.3780 (3)	0.54435 (12)	0.0200 (5)	0.5
H15A	0.232077	0.457762	0.562042	0.024*	0.5
H15B	0.056164	0.348121	0.582008	0.024*	0.5
C16	0.2097 (8)	0.2055 (7)	0.5141 (2)	0.0294 (9)	0.5
H16A	0.287070	0.231030	0.477391	0.044*	0.5
H16B	0.111372	0.120372	0.498353	0.044*	0.5
H16C	0.283592	0.151291	0.546492	0.044*	0.5
N3	0.000000	0.500000	0.000000	0.0202 (3)	
C17	0.1133 (3)	0.6156 (4)	0.04895 (12)	0.0203 (5)	0.5
H17A	0.144339	0.542200	0.084771	0.024*	0.5
H17B	0.038115	0.718759	0.067749	0.024*	0.5
C18	0.2895 (10)	0.6926 (11)	0.0236 (3)	0.0241 (12)	0.5
H18A	0.263217	0.751858	−0.015658	0.036*	0.5
H18B	0.375739	0.593461	0.012908	0.036*	0.5
H18C	0.343016	0.782185	0.057094	0.036*	0.5
C19	0.1230 (4)	0.3261 (4)	−0.02561 (13)	0.0220 (5)	0.5
H19A	0.050750	0.245969	−0.058044	0.026*	0.5
H19B	0.231016	0.370313	−0.048318	0.026*	0.5
C20	0.1878 (9)	0.2147 (8)	0.0260 (2)	0.0339 (11)	0.5
H20A	0.256830	0.110556	0.005803	0.051*	0.5
H20B	0.082425	0.170294	0.049113	0.051*	0.5
H20C	0.266708	0.289971	0.056877	0.051*	0.5
C21	−0.0425 (4)	0.5803 (4)	−0.06067 (13)	0.0248 (5)	0.5
H21A	−0.115866	0.491836	−0.089868	0.030*	0.5
H21B	0.072697	0.605048	−0.083298	0.030*	0.5
C22	−0.1486 (9)	0.7582 (8)	−0.0469 (3)	0.0382 (12)	0.5
H22A	−0.190638	0.798019	−0.088193	0.057*	0.5
H22B	−0.068804	0.852512	−0.024298	0.057*	0.5
H22C	−0.254307	0.737617	−0.019506	0.057*	0.5
C23	−0.1621 (3)	0.4287 (4)	0.03386 (12)	0.0220 (5)	0.5
H23A	−0.236322	0.534030	0.051294	0.026*	0.5
H23B	−0.117317	0.369411	0.071589	0.026*	0.5
C24	−0.2862 (10)	0.2942 (11)	−0.0066 (3)	0.0254 (12)	0.5
H24A	−0.322183	0.345469	−0.046525	0.038*	0.5
H24B	−0.395464	0.270328	0.018617	0.038*	0.5
H24C	−0.221212	0.179936	−0.017931	0.038*	0.5

### Bis(tetraethylammonium) molybdate dihydrate (100k) Atomic displacement parameters (Å^2^)

**Table:**

	*U*^11^	*U*^22^	*U*^33^	*U*^12^	*U*^13^	*U*^23^
Mo1	0.01164 (5)	0.01352 (5)	0.02353 (6)	0.00006 (3)	0.00236 (4)	−0.00176 (4)
O1	0.0177 (4)	0.0272 (5)	0.0390 (6)	0.0007 (4)	0.0093 (4)	−0.0047 (4)
O2	0.0277 (5)	0.0207 (4)	0.0364 (6)	0.0028 (4)	0.0058 (4)	0.0067 (4)
O3	0.0194 (4)	0.0291 (5)	0.0312 (5)	0.0015 (4)	−0.0034 (4)	−0.0011 (4)
O4	0.0251 (5)	0.0217 (4)	0.0308 (5)	−0.0029 (4)	0.0034 (4)	−0.0075 (4)
O1W	0.0326 (5)	0.0242 (5)	0.0212 (5)	−0.0010 (4)	0.0022 (4)	−0.0011 (4)
O2W	0.0264 (5)	0.0350 (5)	0.0195 (5)	0.0078 (4)	0.0008 (4)	0.0027 (4)
N1	0.0128 (4)	0.0151 (4)	0.0253 (5)	0.0005 (4)	0.0033 (4)	0.0009 (4)
C1	0.0161 (6)	0.0243 (6)	0.0344 (7)	0.0060 (5)	0.0024 (5)	0.0033 (5)
C2	0.0377 (9)	0.0444 (10)	0.0606 (12)	0.0140 (8)	0.0073 (8)	0.0298 (9)
C3	0.0168 (6)	0.0197 (6)	0.0352 (7)	−0.0017 (5)	0.0011 (5)	−0.0063 (5)
C4	0.0234 (7)	0.0417 (9)	0.0367 (8)	0.0036 (6)	−0.0065 (6)	−0.0058 (7)
C5	0.0161 (5)	0.0224 (6)	0.0323 (7)	0.0032 (5)	0.0038 (5)	−0.0053 (5)
C6	0.0252 (7)	0.0369 (8)	0.0418 (9)	−0.0005 (6)	0.0039 (6)	−0.0168 (7)
C7	0.0276 (7)	0.0303 (7)	0.0341 (8)	−0.0067 (6)	0.0055 (6)	0.0112 (6)
C8	0.0345 (9)	0.0728 (13)	0.0291 (8)	−0.0068 (8)	0.0093 (7)	0.0096 (8)
N2	0.0174 (7)	0.0741 (14)	0.0121 (7)	−0.0108 (8)	−0.0008 (6)	−0.0018 (8)
C9	0.0256 (13)	0.0314 (13)	0.0145 (11)	−0.0036 (10)	0.0038 (9)	0.0052 (10)
C10	0.0265 (16)	0.037 (2)	0.031 (2)	−0.0065 (16)	0.0077 (15)	0.0086 (15)
C11	0.0251 (12)	0.0207 (12)	0.0193 (12)	−0.0006 (10)	0.0004 (10)	−0.0052 (9)
C12	0.034 (2)	0.032 (2)	0.019 (2)	−0.0031 (15)	−0.0011 (18)	−0.0036 (16)
C13	0.0183 (11)	0.0223 (11)	0.0199 (12)	−0.0036 (9)	−0.0027 (9)	−0.0021 (9)
C14	0.0207 (14)	0.032 (2)	0.031 (2)	−0.0038 (14)	0.0059 (13)	0.0023 (14)
C15	0.0196 (11)	0.0241 (12)	0.0164 (11)	−0.0016 (9)	−0.0023 (9)	0.0027 (9)
C16	0.033 (2)	0.038 (2)	0.016 (2)	−0.0004 (15)	0.0000 (17)	0.0001 (16)
N3	0.0212 (7)	0.0280 (8)	0.0115 (6)	−0.0032 (6)	−0.0001 (5)	0.0028 (6)
C17	0.0215 (11)	0.0244 (12)	0.0140 (11)	−0.0010 (9)	−0.0027 (9)	−0.0018 (9)
C18	0.0258 (18)	0.0294 (18)	0.018 (3)	−0.0054 (13)	−0.0035 (18)	0.006 (2)
C19	0.0212 (12)	0.0215 (11)	0.0227 (12)	0.0045 (9)	0.0013 (9)	−0.0014 (9)
C20	0.039 (3)	0.030 (2)	0.034 (3)	0.0031 (16)	−0.008 (2)	0.011 (2)
C21	0.0321 (14)	0.0267 (13)	0.0166 (12)	−0.0007 (11)	−0.0056 (10)	0.0072 (10)
C22	0.043 (3)	0.034 (2)	0.040 (3)	−0.0001 (19)	−0.019 (2)	0.013 (2)
C23	0.0188 (11)	0.0294 (13)	0.0172 (11)	−0.0018 (10)	0.0040 (9)	0.0004 (9)
C24	0.0204 (17)	0.037 (2)	0.019 (3)	−0.0045 (14)	−0.0046 (18)	0.002 (2)

### Bis(tetraethylammonium) molybdate dihydrate (100k) Geometric parameters (Å, º)

**Table:**

Mo1—O1	1.7636 (9)	C11—H11A	0.9900
Mo1—O2	1.7654 (10)	C11—H11B	0.9900
Mo1—O3	1.7593 (10)	C11—C12	1.536 (5)
Mo1—O4	1.7668 (9)	C12—H12A	0.9800
O1W—H1WA	0.844 (14)	C12—H12B	0.9800
O1W—H1WB	0.818 (14)	C12—H12C	0.9800
O2W—H2WA	0.824 (14)	C13—H13A	0.9900
O2W—H2WB	0.800 (14)	C13—H13B	0.9900
N1—C1	1.5133 (16)	C13—C14	1.512 (4)
N1—C3	1.5145 (16)	C14—H14A	0.9800
N1—C5	1.5145 (16)	C14—H14B	0.9800
N1—C7	1.5117 (17)	C14—H14C	0.9800
C1—H1A	0.9900	C15—H15A	0.9900
C1—H1B	0.9900	C15—H15B	0.9900
C1—C2	1.507 (2)	C15—C16	1.483 (6)
C2—H2A	0.9800	C16—H16A	0.9800
C2—H2B	0.9800	C16—H16B	0.9800
C2—H2C	0.9800	C16—H16C	0.9800
C3—H3A	0.9900	N3—C17	1.491 (2)
C3—H3B	0.9900	N3—C19	1.620 (2)
C3—C4	1.506 (2)	N3—C21	1.475 (3)
C4—H4A	0.9800	N3—C23	1.498 (2)
C4—H4B	0.9800	C17—H17A	0.9900
C4—H4C	0.9800	C17—H17B	0.9900
C5—H5A	0.9900	C17—C18	1.520 (6)
C5—H5B	0.9900	C18—H18A	0.9800
C5—C6	1.515 (2)	C18—H18B	0.9800
C6—H6A	0.9800	C18—H18C	0.9800
C6—H6B	0.9800	C19—H19A	0.9900
C6—H6C	0.9800	C19—H19B	0.9900
C7—H7A	0.9900	C19—C20	1.493 (6)
C7—H7B	0.9900	C20—H20A	0.9800
C7—C8	1.511 (2)	C20—H20B	0.9800
C8—H8A	0.9800	C20—H20C	0.9800
C8—H8B	0.9800	C21—H21A	0.9900
C8—H8C	0.9800	C21—H21B	0.9900
N2—C9	1.479 (2)	C21—C22	1.534 (6)
N2—C11	1.361 (2)	C22—H22A	0.9800
N2—C13	1.617 (2)	C22—H22B	0.9800
N2—C15	1.656 (2)	C22—H22C	0.9800
C9—H9A	0.9900	C23—H23A	0.9900
C9—H9B	0.9900	C23—H23B	0.9900
C9—C10	1.526 (4)	C23—C24	1.515 (6)
C10—H10A	0.9800	C24—H24A	0.9800
C10—H10B	0.9800	C24—H24B	0.9800
C10—H10C	0.9800	C24—H24C	0.9800
			
O1—Mo1—O2	109.45 (5)	C12—C11—H11B	109.3
O1—Mo1—O4	109.38 (5)	C11—C12—H12A	109.5
O2—Mo1—O4	109.27 (5)	C11—C12—H12B	109.5
O3—Mo1—O1	109.36 (5)	C11—C12—H12C	109.5
O3—Mo1—O2	109.50 (5)	H12A—C12—H12B	109.5
O3—Mo1—O4	109.86 (5)	H12A—C12—H12C	109.5
H1WA—O1W—H1WB	104.7 (16)	H12B—C12—H12C	109.5
H2WA—O2W—H2WB	107.2 (17)	N2—C13—H13A	108.4
C1—N1—C3	108.07 (10)	N2—C13—H13B	108.4
C1—N1—C5	111.86 (11)	H13A—C13—H13B	107.5
C3—N1—C5	108.31 (9)	C14—C13—N2	115.5 (2)
C7—N1—C1	108.77 (10)	C14—C13—H13A	108.4
C7—N1—C3	112.11 (11)	C14—C13—H13B	108.4
C7—N1—C5	107.77 (10)	C13—C14—H14A	109.5
N1—C1—H1A	108.6	C13—C14—H14B	109.5
N1—C1—H1B	108.6	C13—C14—H14C	109.5
H1A—C1—H1B	107.6	H14A—C14—H14B	109.5
C2—C1—N1	114.67 (11)	H14A—C14—H14C	109.5
C2—C1—H1A	108.6	H14B—C14—H14C	109.5
C2—C1—H1B	108.6	N2—C15—H15A	107.4
C1—C2—H2A	109.5	N2—C15—H15B	107.4
C1—C2—H2B	109.5	H15A—C15—H15B	106.9
C1—C2—H2C	109.5	C16—C15—N2	119.7 (2)
H2A—C2—H2B	109.5	C16—C15—H15A	107.4
H2A—C2—H2C	109.5	C16—C15—H15B	107.4
H2B—C2—H2C	109.5	C15—C16—H16A	109.5
N1—C3—H3A	108.4	C15—C16—H16B	109.5
N1—C3—H3B	108.4	C15—C16—H16C	109.5
H3A—C3—H3B	107.4	H16A—C16—H16B	109.5
C4—C3—N1	115.61 (11)	H16A—C16—H16C	109.5
C4—C3—H3A	108.4	H16B—C16—H16C	109.5
C4—C3—H3B	108.4	C17—N3—C19	106.60 (14)
C3—C4—H4A	109.5	C17—N3—C23	108.60 (14)
C3—C4—H4B	109.5	C21—N3—C17	115.98 (15)
C3—C4—H4C	109.5	C21—N3—C19	102.74 (15)
H4A—C4—H4B	109.5	C21—N3—C23	114.93 (15)
H4A—C4—H4C	109.5	C23—N3—C19	107.15 (14)
H4B—C4—H4C	109.5	N3—C17—H17A	108.3
N1—C5—H5A	108.6	N3—C17—H17B	108.3
N1—C5—H5B	108.6	N3—C17—C18	115.8 (3)
N1—C5—C6	114.85 (11)	H17A—C17—H17B	107.4
H5A—C5—H5B	107.5	C18—C17—H17A	108.3
C6—C5—H5A	108.6	C18—C17—H17B	108.3
C6—C5—H5B	108.6	C17—C18—H18A	109.5
C5—C6—H6A	109.5	C17—C18—H18B	109.5
C5—C6—H6B	109.5	C17—C18—H18C	109.5
C5—C6—H6C	109.5	H18A—C18—H18B	109.5
H6A—C6—H6B	109.5	H18A—C18—H18C	109.5
H6A—C6—H6C	109.5	H18B—C18—H18C	109.5
H6B—C6—H6C	109.5	N3—C19—H19A	108.4
N1—C7—H7A	108.5	N3—C19—H19B	108.4
N1—C7—H7B	108.5	H19A—C19—H19B	107.5
H7A—C7—H7B	107.5	C20—C19—N3	115.3 (2)
C8—C7—N1	115.12 (13)	C20—C19—H19A	108.4
C8—C7—H7A	108.5	C20—C19—H19B	108.4
C8—C7—H7B	108.5	C19—C20—H20A	109.5
C7—C8—H8A	109.5	C19—C20—H20B	109.5
C7—C8—H8B	109.5	C19—C20—H20C	109.5
C7—C8—H8C	109.5	H20A—C20—H20B	109.5
H8A—C8—H8B	109.5	H20A—C20—H20C	109.5
H8A—C8—H8C	109.5	H20B—C20—H20C	109.5
H8B—C8—H8C	109.5	N3—C21—H21A	109.3
C9—N2—C13	102.45 (14)	N3—C21—H21B	109.3
C9—N2—C15	106.25 (14)	N3—C21—C22	111.6 (3)
C11—N2—C9	124.30 (16)	H21A—C21—H21B	108.0
C11—N2—C13	114.68 (15)	C22—C21—H21A	109.3
C11—N2—C15	106.85 (15)	C22—C21—H21B	109.3
C13—N2—C15	99.32 (13)	C21—C22—H22A	109.5
N2—C9—H9A	109.2	C21—C22—H22B	109.5
N2—C9—H9B	109.2	C21—C22—H22C	109.5
N2—C9—C10	111.9 (2)	H22A—C22—H22B	109.5
H9A—C9—H9B	107.9	H22A—C22—H22C	109.5
C10—C9—H9A	109.2	H22B—C22—H22C	109.5
C10—C9—H9B	109.2	N3—C23—H23A	108.1
C9—C10—H10A	109.5	N3—C23—H23B	108.1
C9—C10—H10B	109.5	N3—C23—C24	116.8 (3)
C9—C10—H10C	109.5	H23A—C23—H23B	107.3
H10A—C10—H10B	109.5	C24—C23—H23A	108.1
H10A—C10—H10C	109.5	C24—C23—H23B	108.1
H10B—C10—H10C	109.5	C23—C24—H24A	109.5
N2—C11—H11A	109.3	C23—C24—H24B	109.5
N2—C11—H11B	109.3	C23—C24—H24C	109.5
N2—C11—C12	111.7 (2)	H24A—C24—H24B	109.5
H11A—C11—H11B	107.9	H24A—C24—H24C	109.5
C12—C11—H11A	109.3	H24B—C24—H24C	109.5
			
C1—N1—C3—C4	−176.43 (12)	C13—N2—C9—C10	−173.6 (2)
C1—N1—C5—C6	51.25 (16)	C13—N2—C11—C12	−67.3 (3)
C1—N1—C7—C8	63.72 (16)	C13—N2—C15—C16	58.8 (3)
C3—N1—C1—C2	−62.38 (17)	C15—N2—C9—C10	−69.9 (3)
C3—N1—C5—C6	170.24 (13)	C15—N2—C11—C12	−176.3 (3)
C3—N1—C7—C8	−55.73 (16)	C15—N2—C13—C14	67.5 (3)
C5—N1—C1—C2	56.76 (17)	C17—N3—C19—C20	57.7 (4)
C5—N1—C3—C4	62.21 (15)	C17—N3—C21—C22	−60.0 (4)
C5—N1—C7—C8	−174.82 (13)	C17—N3—C23—C24	−174.8 (4)
C7—N1—C1—C2	175.68 (14)	C19—N3—C17—C18	62.0 (4)
C7—N1—C3—C4	−56.57 (16)	C19—N3—C21—C22	−175.9 (3)
C7—N1—C5—C6	−68.27 (16)	C19—N3—C23—C24	−60.1 (4)
C9—N2—C11—C12	59.6 (3)	C21—N3—C17—C18	−51.6 (4)
C9—N2—C13—C14	176.5 (3)	C21—N3—C19—C20	−179.8 (4)
C9—N2—C15—C16	−47.2 (3)	C21—N3—C23—C24	53.4 (4)
C11—N2—C9—C10	54.4 (3)	C23—N3—C17—C18	177.2 (4)
C11—N2—C13—C14	−46.0 (3)	C23—N3—C19—C20	−58.4 (4)
C11—N2—C15—C16	178.3 (3)	C23—N3—C21—C22	68.1 (3)

### Bis(tetraethylammonium) molybdate dihydrate (100k) Hydrogen-bond geometry (Å, º)

**Table:**

*D*—H···*A*	*D*—H	H···*A*	*D*···*A*	*D*—H···*A*
O1*W*—H1*WA*···O2	0.85 (2)	1.93 (2)	2.7693 (14)	173 (2)
O1*W*—H1*WB*···O4^i^	0.82 (2)	1.93 (2)	2.7466 (14)	174 (2)
O2*W*—H2*WA*···O1	0.83 (2)	1.93 (2)	2.7539 (15)	177 (2)
O2*W*—H2*WB*···O3^ii^	0.80 (2)	1.95 (2)	2.7462 (15)	177 (2)
C1—H1*A*···O3^iii^	0.99	2.45	3.3193 (17)	146
C1—H1*B*···O1*W*^ii^	0.99	2.52	3.4147 (17)	149
C3—H3*A*···O2*W*^i^	0.99	2.46	3.4043 (17)	158
C3—H3*B*···O4^i^	0.99	2.39	3.3081 (17)	154
C4—H4*B*···O2	0.98	2.51	3.4881 (18)	173
C5—H5*A*···O1*W*	0.99	2.59	3.4059 (17)	140
C7—H7*B*···O2^ii^	0.99	2.59	3.5499 (18)	163
C9—H9*A*···O4	0.99	2.29	3.057 (3)	134
C12—H12*B*···O1*W*^iv^	0.98	2.50	3.159 (5)	124
C13—H13*B*···O1*W*	0.99	2.51	3.461 (3)	162
C15—H15*B*···O4^iv^	0.99	2.48	3.432 (3)	162
C21—H21*B*···O3^v^	0.99	2.46	3.168 (3)	128

Symmetry codes: (i) *x*, *y*−1, *z*; (ii) *x*+1, *y*, *z*; (iii) *x*+1, *y*−1, *z*; (iv) −*x*, −*y*+1, −*z*+1; (v) −*x*, −*y*+1, −*z*.

### Bis(tetraethylammonium) molybdate dihydrate (200k) Crystal data

**Table:**

(C_8_H_20_N)_2_[MoO_4_]·2H_2_O	*Z* = 4
*M**_r_* = 456.47	*F*(000) = 976
Triclinic, *P*1	*D*_x_ = 1.351 Mg m^−^^3^
*a* = 7.4299 (2) Å	Mo *K*α radiation, λ = 0.71073 Å
*b* = 14.7269 (4) Å	Cell parameters from 9701 reflections
*c* = 20.6213 (7) Å	θ = 2.9–27.5°
α = 90.065 (1)°	µ = 0.61 mm^−^^1^
β = 95.821 (1)°	*T* = 200 K
γ = 90.898 (1)°	Plate, colourless
*V* = 2244.45 (11) Å^3^	0.15 × 0.15 × 0.01 mm

### Bis(tetraethylammonium) molybdate dihydrate (200k) Data collection

**Table:**

Bruker D8 VENTURE diffractometer	10335 independent reflections
Radiation source: microfocus sealed tube, Incoatec IµS	8514 reflections with *I* > 2σ(*I*)
Multilayer mirror monochromator	*R*_int_ = 0.055
φ and ω scans	θ_max_ = 27.5°, θ_min_ = 2.4°
Absorption correction: multi-scan (SADABS; Krause *et al.*, 2015)	*h* = −9→9
*T*_min_ = 0.710, *T*_max_ = 0.746	*k* = −19→19
95151 measured reflections	*l* = −26→26

### Bis(tetraethylammonium) molybdate dihydrate (200k) Refinement

**Table:**

Refinement on *F*^2^	Primary atom site location: dual
Least-squares matrix: full	Hydrogen site location: mixed
*R*[*F*^2^ > 2σ(*F*^2^)] = 0.028	H atoms treated by a mixture of independent and constrained refinement
*wR*(*F*^2^) = 0.064	*w* = 1/[σ^2^(*F*_o_^2^) + (0.0181*P*)^2^ + 1.7213*P*] where *P* = (*F*_o_^2^ + 2*F*_c_^2^)/3
*S* = 1.04	(Δ/σ)_max_ = 0.001
10335 reflections	Δρ_max_ = 0.33 e Å^−^^3^
659 parameters	Δρ_min_ = −0.45 e Å^−^^3^
912 restraints	

### Bis(tetraethylammonium) molybdate dihydrate (200k) Special details

**Table:**

Geometry. All esds (except the esd in the dihedral angle between two l.s. planes) are estimated using the full covariance matrix. The cell esds are taken into account individually in the estimation of esds in distances, angles and torsion angles; correlations between esds in cell parameters are only used when they are defined by crystal symmetry. An approximate (isotropic) treatment of cell esds is used for estimating esds involving l.s. planes.

### Bis(tetraethylammonium) molybdate dihydrate (200k) Fractional atomic coordinates and isotropic or equivalent isotropic displacement parameters (Å^2^)

**Table:**

	*x*	*y*	*z*	*U*_iso_*/*U*_eq_	Occ. (<1)
Mo1	0.54730 (2)	0.73426 (2)	0.24651 (2)	0.02131 (5)	
O1	0.5066 (2)	0.82584 (11)	0.29719 (8)	0.0401 (4)	
O2	0.7418 (2)	0.75794 (11)	0.20696 (8)	0.0395 (4)	
O3	0.3610 (2)	0.71645 (12)	0.18784 (8)	0.0400 (4)	
O4	0.5835 (2)	0.63617 (10)	0.29408 (8)	0.0361 (4)	
Mo2	0.52665 (2)	0.23447 (2)	0.25351 (2)	0.02095 (5)	
O5	0.5113 (2)	0.33000 (11)	0.30354 (9)	0.0448 (4)	
O6	0.7123 (2)	0.24759 (12)	0.20782 (9)	0.0411 (4)	
O7	0.32790 (19)	0.22295 (11)	0.19990 (8)	0.0338 (4)	
O8	0.5536 (2)	0.13681 (10)	0.30262 (8)	0.0363 (4)	
O1W	0.5005 (2)	0.97837 (12)	0.37273 (8)	0.0362 (4)	
H1WA	0.508 (4)	0.9349 (13)	0.3482 (12)	0.054*	
H1WB	0.522 (4)	1.0243 (12)	0.3517 (12)	0.054*	
O2W	0.5327 (2)	0.49132 (11)	0.37260 (8)	0.0364 (4)	
H2WA	0.551 (4)	0.5337 (13)	0.3482 (12)	0.055*	
H2WB	0.531 (4)	0.4443 (12)	0.3522 (12)	0.055*	
O3W	0.0168 (2)	0.73959 (13)	0.12795 (8)	0.0407 (4)	
H3WA	0.115 (2)	0.733 (2)	0.1493 (13)	0.061*	
H3WB	−0.061 (3)	0.744 (2)	0.1546 (12)	0.061*	
O4W	−0.0055 (2)	0.24150 (12)	0.12855 (8)	0.0365 (4)	
H4WA	0.094 (2)	0.2328 (19)	0.1491 (12)	0.055*	
H4WB	−0.082 (3)	0.2446 (19)	0.1549 (12)	0.055*	
N1	0.0283 (2)	0.49515 (11)	0.25153 (8)	0.0235 (3)	
C1	0.1567 (3)	0.56867 (14)	0.28064 (11)	0.0306 (5)	
H1A	0.250269	0.539803	0.311125	0.037*	
H1B	0.218846	0.596347	0.245082	0.037*	
C2	0.0721 (4)	0.64337 (18)	0.31649 (13)	0.0452 (6)	
H2A	−0.023280	0.671675	0.287358	0.068*	
H2B	0.164712	0.689121	0.331031	0.068*	
H2C	0.019825	0.617943	0.354408	0.068*	
C3	−0.0754 (3)	0.45023 (18)	0.30243 (13)	0.0427 (6)	
H3A	−0.151617	0.400591	0.281379	0.051*	
H3B	−0.157331	0.495352	0.318814	0.051*	
C4	0.0415 (4)	0.4115 (2)	0.36006 (14)	0.0622 (8)	
H4A	0.127409	0.368802	0.344382	0.093*	
H4B	−0.035438	0.379632	0.388851	0.093*	
H4C	0.108132	0.460910	0.384169	0.093*	
C5	0.1398 (3)	0.42391 (15)	0.22177 (12)	0.0357 (5)	
H5A	0.230343	0.400924	0.256132	0.043*	
H5B	0.059027	0.372312	0.206856	0.043*	
C6	0.2370 (4)	0.4560 (2)	0.16549 (15)	0.0641 (9)	
H6A	0.148547	0.475839	0.130058	0.096*	
H6B	0.307673	0.406166	0.150080	0.096*	
H6C	0.318220	0.506884	0.179586	0.096*	
C7	−0.1063 (3)	0.53824 (15)	0.20142 (12)	0.0353 (5)	
H7A	−0.181479	0.580470	0.224050	0.042*	
H7B	−0.038819	0.574660	0.171297	0.042*	
C8	−0.2298 (4)	0.47221 (19)	0.16177 (16)	0.0631 (9)	
H8A	−0.312089	0.505756	0.130649	0.095*	
H8B	−0.300285	0.436919	0.190870	0.095*	
H8C	−0.157281	0.431050	0.138032	0.095*	
N2	0.0072 (2)	−0.00407 (11)	0.25172 (8)	0.0221 (3)	
C9	0.1260 (3)	0.06949 (14)	0.28603 (11)	0.0286 (5)	
H9A	0.207575	0.040910	0.320842	0.034*	
H9B	0.202680	0.096513	0.254289	0.034*	
C10	0.0267 (3)	0.14477 (17)	0.31590 (12)	0.0412 (6)	
H10A	−0.054682	0.173836	0.282021	0.062*	
H10B	0.114206	0.189820	0.335635	0.062*	
H10C	−0.043905	0.119618	0.349480	0.062*	
C11	−0.1208 (3)	−0.04618 (17)	0.29598 (12)	0.0365 (5)	
H11A	−0.206073	0.000823	0.307457	0.044*	
H11B	−0.192669	−0.094564	0.271502	0.044*	
C12	−0.0315 (4)	−0.0863 (2)	0.35813 (13)	0.0521 (7)	
H12A	0.051201	−0.134085	0.347548	0.078*	
H12B	−0.124176	−0.112236	0.383540	0.078*	
H12C	0.036676	−0.038657	0.383697	0.078*	
C13	0.1292 (3)	−0.07716 (14)	0.23013 (11)	0.0326 (5)	
H13A	0.202042	−0.101064	0.269069	0.039*	
H13B	0.052680	−0.127793	0.210656	0.039*	
C14	0.2557 (4)	−0.04641 (19)	0.18162 (14)	0.0530 (7)	
H14A	0.185154	−0.023951	0.142324	0.079*	
H14B	0.328969	−0.097627	0.170118	0.079*	
H14C	0.335177	0.002349	0.200839	0.079*	
C15	−0.1054 (3)	0.03809 (15)	0.19470 (11)	0.0336 (5)	
H15A	−0.024061	0.073700	0.168879	0.040*	
H15B	−0.190417	0.080993	0.211834	0.040*	
C16	−0.2132 (4)	−0.02871 (18)	0.14964 (13)	0.0504 (7)	
H16A	−0.290118	0.004600	0.116667	0.076*	
H16B	−0.289121	−0.067042	0.174990	0.076*	
H16C	−0.130119	−0.066914	0.128127	0.076*	
N3	0.5132 (3)	0.24560 (15)	0.50052 (11)	0.0249 (4)	0.938 (2)
N4	0.5236 (3)	0.24750 (19)	0.00038 (14)	0.0275 (6)	0.884 (2)
C17	0.5723 (3)	0.32998 (17)	0.46553 (11)	0.0356 (6)	0.938 (2)
H17A	0.628431	0.310637	0.426341	0.043*	0.938 (2)
H17B	0.463094	0.364838	0.450470	0.043*	0.938 (2)
C18	0.7036 (5)	0.3926 (3)	0.50517 (17)	0.0454 (9)	0.938 (2)
H18A	0.732501	0.444896	0.478587	0.068*	0.938 (2)
H18B	0.814737	0.359796	0.518984	0.068*	0.938 (2)
H18C	0.648951	0.413588	0.543678	0.068*	0.938 (2)
C19	0.4230 (3)	0.27013 (18)	0.56043 (11)	0.0351 (6)	0.938 (2)
H19A	0.509339	0.306882	0.589754	0.042*	0.938 (2)
H19B	0.396705	0.213492	0.583756	0.042*	0.938 (2)
C20	0.2488 (4)	0.3224 (3)	0.54744 (17)	0.0541 (9)	0.938 (2)
H20A	0.270952	0.376849	0.521953	0.081*	0.938 (2)
H20B	0.206145	0.340270	0.588981	0.081*	0.938 (2)
H20C	0.156766	0.283908	0.523007	0.081*	0.938 (2)
C21	0.6741 (3)	0.18822 (17)	0.52477 (12)	0.0333 (5)	0.938 (2)
H21A	0.754029	0.224308	0.556760	0.040*	0.938 (2)
H21B	0.629675	0.134807	0.547915	0.040*	0.938 (2)
C22	0.7850 (4)	0.1552 (2)	0.47208 (16)	0.0506 (8)	0.938 (2)
H22A	0.707116	0.119974	0.439718	0.076*	0.938 (2)
H22B	0.882425	0.116741	0.491553	0.076*	0.938 (2)
H22C	0.837194	0.207436	0.450843	0.076*	0.938 (2)
C23	0.3861 (3)	0.19281 (17)	0.45159 (11)	0.0313 (5)	0.938 (2)
H23A	0.284990	0.232605	0.435665	0.038*	0.938 (2)
H23B	0.452160	0.177447	0.413789	0.038*	0.938 (2)
C24	0.3083 (6)	0.1065 (2)	0.47729 (15)	0.0376 (8)	0.938 (2)
H24A	0.226476	0.077472	0.442849	0.056*	0.938 (2)
H24B	0.241258	0.120707	0.514462	0.056*	0.938 (2)
H24C	0.406748	0.065101	0.491273	0.056*	0.938 (2)
C25	0.6466 (3)	0.32600 (17)	0.02515 (13)	0.0340 (6)	0.884 (2)
H25A	0.581484	0.363644	0.054683	0.041*	0.884 (2)
H25B	0.753918	0.301106	0.051143	0.041*	0.884 (2)
C26	0.7104 (5)	0.3863 (3)	−0.02722 (18)	0.0503 (9)	0.884 (2)
H26A	0.771705	0.349565	−0.057716	0.076*	0.884 (2)
H26B	0.794770	0.432555	−0.007236	0.076*	0.884 (2)
H26C	0.606243	0.415957	−0.050759	0.076*	0.884 (2)
C27	0.4713 (4)	0.1999 (2)	0.06110 (13)	0.0435 (7)	0.884 (2)
H27A	0.583104	0.181377	0.087685	0.052*	0.884 (2)
H27B	0.408991	0.243730	0.087189	0.052*	0.884 (2)
C28	0.3497 (6)	0.1168 (3)	0.0481 (2)	0.0611 (12)	0.884 (2)
H28A	0.244510	0.132671	0.017965	0.092*	0.884 (2)
H28B	0.308983	0.095234	0.089241	0.092*	0.884 (2)
H28C	0.417128	0.068759	0.028834	0.092*	0.884 (2)
C29	0.6210 (4)	0.18454 (17)	−0.04176 (12)	0.0331 (6)	0.884 (2)
H29A	0.536571	0.134673	−0.057288	0.040*	0.884 (2)
H29B	0.651906	0.218697	−0.080558	0.040*	0.884 (2)
C30	0.7912 (6)	0.1434 (3)	−0.00951 (18)	0.0476 (10)	0.884 (2)
H30A	0.848383	0.107249	−0.041470	0.071*	0.884 (2)
H30B	0.761326	0.104182	0.026383	0.071*	0.884 (2)
H30C	0.874870	0.191810	0.007564	0.071*	0.884 (2)
C31	0.3591 (4)	0.28052 (19)	−0.04168 (13)	0.0380 (6)	0.884 (2)
H31A	0.283224	0.227231	−0.056684	0.046*	0.884 (2)
H31B	0.399860	0.310345	−0.080762	0.046*	0.884 (2)
C32	0.2435 (5)	0.3463 (3)	−0.00837 (19)	0.0548 (10)	0.884 (2)
H32A	0.138252	0.362597	−0.038399	0.082*	0.884 (2)
H32B	0.314860	0.401221	0.004278	0.082*	0.884 (2)
H32C	0.202583	0.317779	0.030572	0.082*	0.884 (2)
N3B	0.504 (3)	0.2554 (19)	0.4998 (14)	0.032 (4)	0.062 (2)
C17B	0.624 (5)	0.310 (2)	0.5502 (13)	0.032 (4)	0.062 (2)
H17C	0.547771	0.335192	0.582318	0.038*	0.062 (2)
H17D	0.711153	0.268542	0.573777	0.038*	0.062 (2)
C18B	0.729 (9)	0.387 (4)	0.523 (3)	0.038 (6)	0.062 (2)
H18D	0.802476	0.418491	0.559346	0.057*	0.062 (2)
H18E	0.808937	0.363714	0.492575	0.057*	0.062 (2)
H18F	0.645149	0.430530	0.501136	0.057*	0.062 (2)
C19B	0.341 (4)	0.311 (3)	0.4727 (13)	0.036 (4)	0.062 (2)
H19C	0.386039	0.366446	0.452390	0.044*	0.062 (2)
H19D	0.271256	0.274875	0.437751	0.044*	0.062 (2)
C20B	0.215 (5)	0.338 (4)	0.5213 (19)	0.036 (6)	0.062 (2)
H20D	0.115157	0.373111	0.499398	0.055*	0.062 (2)
H20E	0.165419	0.283618	0.540871	0.055*	0.062 (2)
H20F	0.280500	0.375426	0.555548	0.055*	0.062 (2)
C21B	0.588 (4)	0.233 (3)	0.4376 (13)	0.035 (4)	0.062 (2)
H21C	0.499130	0.197157	0.408854	0.042*	0.062 (2)
H21D	0.609850	0.291013	0.414836	0.042*	0.062 (2)
C22B	0.763 (5)	0.182 (3)	0.446 (2)	0.035 (6)	0.062 (2)
H22D	0.805276	0.171561	0.403225	0.053*	0.062 (2)
H22E	0.853927	0.218002	0.473135	0.053*	0.062 (2)
H22F	0.742876	0.123866	0.467135	0.053*	0.062 (2)
C23B	0.441 (5)	0.174 (2)	0.5370 (13)	0.032 (4)	0.062 (2)
H23C	0.549217	0.141352	0.556427	0.039*	0.062 (2)
H23D	0.376040	0.195889	0.573416	0.039*	0.062 (2)
C24B	0.320 (9)	0.106 (3)	0.498 (2)	0.031 (6)	0.062 (2)
H24D	0.287447	0.056729	0.526390	0.047*	0.062 (2)
H24E	0.209886	0.136771	0.479670	0.047*	0.062 (2)
H24F	0.383556	0.082079	0.462633	0.047*	0.062 (2)
N4B	0.478 (2)	0.2506 (12)	0.0024 (10)	0.031 (4)	0.116 (2)
C25B	0.512 (3)	0.2998 (12)	−0.0598 (8)	0.038 (3)	0.116 (2)
H25C	0.562584	0.256228	−0.089455	0.045*	0.116 (2)
H25D	0.394742	0.320302	−0.081497	0.045*	0.116 (2)
C26B	0.639 (4)	0.3812 (15)	−0.0506 (12)	0.040 (5)	0.116 (2)
H26D	0.719058	0.382606	−0.085637	0.060*	0.116 (2)
H26E	0.712418	0.376653	−0.008429	0.060*	0.116 (2)
H26F	0.568882	0.436975	−0.051697	0.060*	0.116 (2)
C27B	0.352 (3)	0.1709 (13)	−0.0228 (9)	0.037 (3)	0.116 (2)
H27C	0.245905	0.195710	−0.049710	0.045*	0.116 (2)
H27D	0.417534	0.131964	−0.051374	0.045*	0.116 (2)
C28B	0.286 (4)	0.113 (2)	0.0303 (13)	0.046 (5)	0.116 (2)
H28D	0.223444	0.059060	0.011079	0.068*	0.116 (2)
H28E	0.201706	0.148251	0.053819	0.068*	0.116 (2)
H28F	0.388924	0.095142	0.060616	0.068*	0.116 (2)
C29B	0.645 (2)	0.2165 (13)	0.0431 (8)	0.032 (3)	0.116 (2)
H29C	0.719761	0.269758	0.059101	0.039*	0.116 (2)
H29D	0.605780	0.184924	0.081782	0.039*	0.116 (2)
C30B	0.763 (4)	0.153 (2)	0.0095 (13)	0.039 (5)	0.116 (2)
H30D	0.792475	0.180949	−0.031367	0.058*	0.116 (2)
H30E	0.697707	0.095708	0.000152	0.058*	0.116 (2)
H30F	0.874169	0.142508	0.037814	0.058*	0.116 (2)
C31B	0.380 (2)	0.3109 (13)	0.0467 (8)	0.034 (3)	0.116 (2)
H31C	0.353771	0.274971	0.085267	0.041*	0.116 (2)
H31D	0.463228	0.361266	0.062388	0.041*	0.116 (2)
C32B	0.206 (3)	0.3511 (19)	0.0170 (11)	0.039 (5)	0.116 (2)
H32D	0.229015	0.385482	−0.022101	0.059*	0.116 (2)
H32E	0.158850	0.391854	0.048591	0.059*	0.116 (2)
H32F	0.117902	0.302346	0.005178	0.059*	0.116 (2)

### Bis(tetraethylammonium) molybdate dihydrate (200k) Atomic displacement parameters (Å^2^)

**Table:**

	*U*^11^	*U*^22^	*U*^33^	*U*^12^	*U*^13^	*U*^23^
Mo1	0.01899 (8)	0.01799 (9)	0.02674 (10)	−0.00085 (6)	0.00142 (7)	0.00328 (7)
O1	0.0459 (10)	0.0300 (9)	0.0447 (10)	0.0031 (7)	0.0062 (8)	−0.0057 (7)
O2	0.0306 (8)	0.0388 (9)	0.0511 (10)	−0.0007 (7)	0.0148 (8)	0.0085 (8)
O3	0.0329 (8)	0.0445 (10)	0.0399 (10)	−0.0072 (7)	−0.0083 (7)	0.0059 (8)
O4	0.0382 (9)	0.0264 (8)	0.0433 (10)	−0.0012 (7)	0.0021 (7)	0.0116 (7)
Mo2	0.01780 (8)	0.01892 (9)	0.02597 (10)	0.00039 (6)	0.00137 (7)	0.00263 (7)
O5	0.0574 (11)	0.0299 (9)	0.0460 (10)	0.0024 (8)	−0.0007 (9)	−0.0113 (8)
O6	0.0269 (8)	0.0476 (10)	0.0508 (11)	0.0059 (7)	0.0128 (7)	0.0167 (8)
O7	0.0243 (7)	0.0410 (9)	0.0348 (9)	0.0001 (7)	−0.0025 (6)	0.0032 (7)
O8	0.0325 (8)	0.0298 (9)	0.0458 (10)	0.0000 (7)	−0.0004 (7)	0.0141 (7)
O1W	0.0478 (10)	0.0337 (9)	0.0284 (9)	0.0048 (8)	0.0087 (7)	0.0027 (7)
O2W	0.0514 (10)	0.0271 (9)	0.0309 (9)	0.0010 (8)	0.0057 (8)	0.0041 (7)
O3W	0.0296 (8)	0.0596 (12)	0.0326 (9)	−0.0004 (8)	0.0026 (7)	−0.0021 (8)
O4W	0.0304 (8)	0.0485 (10)	0.0299 (9)	0.0004 (8)	−0.0004 (7)	0.0021 (8)
N1	0.0204 (8)	0.0201 (9)	0.0299 (9)	0.0012 (6)	0.0024 (7)	0.0015 (7)
C1	0.0216 (10)	0.0275 (11)	0.0414 (13)	−0.0034 (8)	−0.0015 (9)	−0.0021 (10)
C2	0.0457 (14)	0.0413 (15)	0.0470 (15)	0.0035 (11)	−0.0039 (12)	−0.0158 (12)
C3	0.0365 (13)	0.0449 (15)	0.0486 (15)	−0.0104 (11)	0.0141 (11)	0.0067 (12)
C4	0.083 (2)	0.063 (2)	0.0406 (16)	−0.0122 (17)	0.0089 (15)	0.0187 (14)
C5	0.0392 (12)	0.0246 (12)	0.0443 (14)	0.0101 (9)	0.0075 (11)	−0.0013 (10)
C6	0.077 (2)	0.0600 (19)	0.063 (2)	0.0177 (16)	0.0399 (17)	−0.0018 (15)
C7	0.0344 (12)	0.0255 (12)	0.0431 (14)	0.0047 (9)	−0.0113 (10)	0.0004 (10)
C8	0.0636 (19)	0.0431 (17)	0.073 (2)	−0.0040 (14)	−0.0382 (17)	0.0011 (15)
N2	0.0199 (8)	0.0192 (9)	0.0268 (9)	−0.0013 (6)	0.0003 (7)	0.0024 (7)
C9	0.0209 (9)	0.0250 (11)	0.0388 (12)	−0.0029 (8)	−0.0018 (9)	−0.0014 (9)
C10	0.0406 (13)	0.0387 (14)	0.0422 (14)	0.0042 (11)	−0.0065 (11)	−0.0150 (11)
C11	0.0270 (11)	0.0435 (14)	0.0391 (13)	−0.0112 (10)	0.0059 (10)	0.0093 (11)
C12	0.0586 (17)	0.0609 (18)	0.0364 (14)	−0.0164 (14)	0.0055 (13)	0.0160 (13)
C13	0.0394 (12)	0.0208 (11)	0.0382 (13)	0.0074 (9)	0.0058 (10)	0.0024 (9)
C14	0.0623 (18)	0.0458 (16)	0.0564 (18)	0.0183 (13)	0.0312 (15)	0.0030 (13)
C15	0.0373 (12)	0.0240 (11)	0.0370 (13)	0.0024 (9)	−0.0085 (10)	0.0046 (9)
C16	0.0632 (17)	0.0372 (15)	0.0440 (15)	−0.0034 (13)	−0.0266 (13)	0.0058 (12)
N3	0.0275 (10)	0.0307 (11)	0.0166 (9)	0.0037 (8)	0.0011 (8)	0.0011 (8)
N4	0.0351 (14)	0.0286 (12)	0.0183 (11)	−0.0043 (10)	0.0012 (10)	−0.0002 (9)
C17	0.0444 (14)	0.0362 (14)	0.0258 (12)	−0.0025 (11)	0.0014 (10)	0.0092 (10)
C18	0.052 (2)	0.0382 (17)	0.044 (2)	−0.0089 (15)	−0.0059 (17)	0.0053 (16)
C19	0.0348 (12)	0.0483 (15)	0.0229 (12)	−0.0004 (11)	0.0063 (10)	−0.0077 (10)
C20	0.0415 (17)	0.066 (2)	0.055 (2)	0.0122 (15)	0.0072 (16)	−0.024 (2)
C21	0.0286 (11)	0.0377 (14)	0.0330 (13)	0.0081 (10)	−0.0013 (10)	0.0043 (10)
C22	0.0347 (15)	0.063 (2)	0.055 (2)	0.0106 (14)	0.0076 (15)	−0.0117 (17)
C23	0.0321 (12)	0.0403 (14)	0.0207 (11)	0.0011 (10)	−0.0017 (9)	−0.0034 (10)
C24	0.0370 (15)	0.0389 (15)	0.0359 (18)	−0.0027 (12)	−0.0011 (17)	−0.0027 (14)
C25	0.0367 (13)	0.0297 (13)	0.0338 (14)	−0.0048 (11)	−0.0041 (11)	−0.0083 (11)
C26	0.057 (2)	0.0370 (18)	0.058 (2)	−0.0121 (16)	0.0116 (18)	−0.0024 (17)
C27	0.0617 (18)	0.0441 (16)	0.0262 (14)	−0.0082 (14)	0.0136 (13)	0.0041 (12)
C28	0.073 (3)	0.053 (2)	0.059 (3)	−0.020 (2)	0.024 (2)	0.007 (2)
C29	0.0445 (15)	0.0305 (13)	0.0244 (12)	−0.0016 (11)	0.0051 (11)	−0.0029 (10)
C30	0.053 (2)	0.048 (2)	0.042 (2)	0.0087 (16)	0.0022 (18)	−0.0057 (19)
C31	0.0374 (14)	0.0436 (16)	0.0309 (14)	−0.0016 (12)	−0.0058 (11)	−0.0060 (12)
C32	0.0425 (19)	0.065 (2)	0.055 (3)	0.0113 (16)	−0.0078 (18)	−0.018 (2)
N3B	0.031 (7)	0.038 (7)	0.025 (7)	0.008 (7)	−0.002 (7)	0.000 (7)
C17B	0.034 (7)	0.035 (7)	0.027 (7)	0.002 (7)	0.004 (7)	0.000 (7)
C18B	0.047 (11)	0.027 (11)	0.038 (12)	−0.009 (11)	0.000 (11)	0.008 (11)
C19B	0.038 (7)	0.044 (8)	0.028 (7)	0.009 (7)	0.002 (7)	−0.001 (7)
C20B	0.032 (11)	0.052 (11)	0.029 (11)	0.014 (10)	0.019 (10)	−0.008 (11)
C21B	0.035 (7)	0.043 (8)	0.028 (7)	0.010 (7)	0.003 (7)	−0.003 (7)
C22B	0.032 (10)	0.040 (11)	0.035 (11)	0.015 (10)	0.015 (10)	0.000 (10)
C23B	0.030 (7)	0.039 (7)	0.027 (7)	0.001 (7)	−0.001 (7)	−0.002 (7)
C24B	0.027 (11)	0.037 (11)	0.029 (12)	−0.008 (10)	0.002 (11)	−0.006 (11)
N4B	0.042 (6)	0.028 (6)	0.023 (6)	−0.007 (6)	0.006 (6)	−0.012 (6)
C25B	0.049 (7)	0.032 (6)	0.033 (6)	−0.006 (6)	0.007 (6)	−0.003 (6)
C26B	0.052 (10)	0.025 (8)	0.043 (10)	−0.014 (8)	0.006 (8)	0.009 (8)
C27B	0.049 (7)	0.031 (7)	0.032 (7)	−0.005 (6)	0.001 (6)	0.000 (6)
C28B	0.049 (10)	0.046 (10)	0.040 (10)	−0.016 (9)	−0.007 (9)	0.013 (9)
C29B	0.042 (6)	0.032 (6)	0.022 (6)	−0.003 (6)	−0.003 (6)	−0.011 (6)
C30B	0.046 (10)	0.036 (10)	0.035 (10)	−0.001 (8)	0.015 (9)	−0.004 (9)
C31B	0.043 (7)	0.037 (7)	0.022 (6)	−0.007 (6)	−0.003 (6)	−0.007 (6)
C32B	0.057 (11)	0.035 (10)	0.027 (10)	0.011 (9)	0.004 (9)	0.000 (9)

### Bis(tetraethylammonium) molybdate dihydrate (200k) Geometric parameters (Å, º)

**Table:**

Mo1—O1	1.7540 (16)	C21—H21A	0.9900
Mo1—O2	1.7602 (15)	C21—H21B	0.9900
Mo1—O3	1.7598 (15)	C21—C22	1.513 (4)
Mo1—O4	1.7560 (15)	C22—H22A	0.9800
Mo2—O5	1.7555 (16)	C22—H22B	0.9800
Mo2—O6	1.7559 (15)	C22—H22C	0.9800
Mo2—O7	1.7584 (15)	C23—H23A	0.9900
Mo2—O8	1.7623 (15)	C23—H23B	0.9900
O1W—H1WA	0.822 (16)	C23—C24	1.507 (4)
O1W—H1WB	0.826 (16)	C24—H24A	0.9800
O2W—H2WA	0.821 (16)	C24—H24B	0.9800
O2W—H2WB	0.809 (16)	C24—H24C	0.9800
O3W—H3WA	0.820 (16)	C25—H25A	0.9900
O3W—H3WB	0.837 (16)	C25—H25B	0.9900
O4W—H4WA	0.827 (16)	C25—C26	1.507 (4)
O4W—H4WB	0.827 (16)	C26—H26A	0.9800
N1—C1	1.514 (3)	C26—H26B	0.9800
N1—C3	1.511 (3)	C26—H26C	0.9800
N1—C5	1.514 (3)	C27—H27A	0.9900
N1—C7	1.510 (3)	C27—H27B	0.9900
C1—H1A	0.9900	C27—C28	1.516 (5)
C1—H1B	0.9900	C28—H28A	0.9800
C1—C2	1.505 (3)	C28—H28B	0.9800
C2—H2A	0.9800	C28—H28C	0.9800
C2—H2B	0.9800	C29—H29A	0.9900
C2—H2C	0.9800	C29—H29B	0.9900
C3—H3A	0.9900	C29—C30	1.505 (4)
C3—H3B	0.9900	C30—H30A	0.9800
C3—C4	1.517 (4)	C30—H30B	0.9800
C4—H4A	0.9800	C30—H30C	0.9800
C4—H4B	0.9800	C31—H31A	0.9900
C4—H4C	0.9800	C31—H31B	0.9900
C5—H5A	0.9900	C31—C32	1.514 (4)
C5—H5B	0.9900	C32—H32A	0.9800
C5—C6	1.500 (3)	C32—H32B	0.9800
C6—H6A	0.9800	C32—H32C	0.9800
C6—H6B	0.9800	N3B—C17B	1.524 (15)
C6—H6C	0.9800	N3B—C19B	1.528 (15)
C7—H7A	0.9900	N3B—C21B	1.520 (14)
C7—H7B	0.9900	N3B—C23B	1.522 (15)
C7—C8	1.508 (3)	C17B—H17C	0.9900
C8—H8A	0.9800	C17B—H17D	0.9900
C8—H8B	0.9800	C17B—C18B	1.506 (15)
C8—H8C	0.9800	C18B—H18D	0.9800
N2—C9	1.515 (2)	C18B—H18E	0.9800
N2—C11	1.510 (2)	C18B—H18F	0.9800
N2—C13	1.513 (3)	C19B—H19C	0.9900
N2—C15	1.512 (3)	C19B—H19D	0.9900
C9—H9A	0.9900	C19B—C20B	1.502 (15)
C9—H9B	0.9900	C20B—H20D	0.9800
C9—C10	1.506 (3)	C20B—H20E	0.9800
C10—H10A	0.9800	C20B—H20F	0.9800
C10—H10B	0.9800	C21B—H21C	0.9900
C10—H10C	0.9800	C21B—H21D	0.9900
C11—H11A	0.9900	C21B—C22B	1.506 (15)
C11—H11B	0.9900	C22B—H22D	0.9800
C11—C12	1.508 (3)	C22B—H22E	0.9800
C12—H12A	0.9800	C22B—H22F	0.9800
C12—H12B	0.9800	C23B—H23C	0.9900
C12—H12C	0.9800	C23B—H23D	0.9900
C13—H13A	0.9900	C23B—C24B	1.502 (15)
C13—H13B	0.9900	C24B—H24D	0.9800
C13—C14	1.506 (3)	C24B—H24E	0.9800
C14—H14A	0.9800	C24B—H24F	0.9800
C14—H14B	0.9800	N4B—C25B	1.517 (13)
C14—H14C	0.9800	N4B—C27B	1.55 (3)
C15—H15A	0.9900	N4B—C29B	1.521 (13)
C15—H15B	0.9900	N4B—C31B	1.517 (13)
C15—C16	1.515 (3)	C25B—H25C	0.9900
C16—H16A	0.9800	C25B—H25D	0.9900
C16—H16B	0.9800	C25B—C26B	1.514 (13)
C16—H16C	0.9800	C26B—H26D	0.9800
N3—C17	1.519 (3)	C26B—H26E	0.9800
N3—C19	1.510 (3)	C26B—H26F	0.9800
N3—C21	1.518 (3)	C27B—H27C	0.9900
N3—C23	1.515 (3)	C27B—H27D	0.9900
N4—C25	1.518 (4)	C27B—C28B	1.503 (14)
N4—C27	1.518 (3)	C28B—H28D	0.9800
N4—C29	1.512 (4)	C28B—H28E	0.9800
N4—C31	1.512 (3)	C28B—H28F	0.9800
C17—H17A	0.9900	C29B—H29C	0.9900
C17—H17B	0.9900	C29B—H29D	0.9900
C17—C18	1.509 (4)	C29B—C30B	1.500 (13)
C18—H18A	0.9800	C30B—H30D	0.9800
C18—H18B	0.9800	C30B—H30E	0.9800
C18—H18C	0.9800	C30B—H30F	0.9800
C19—H19A	0.9900	C31B—H31C	0.9900
C19—H19B	0.9900	C31B—H31D	0.9900
C19—C20	1.517 (4)	C31B—C32B	1.502 (14)
C20—H20A	0.9800	C32B—H32D	0.9800
C20—H20B	0.9800	C32B—H32E	0.9800
C20—H20C	0.9800	C32B—H32F	0.9800
			
O1—Mo1—O2	109.38 (8)	H22A—C22—H22C	109.5
O1—Mo1—O3	110.09 (8)	H22B—C22—H22C	109.5
O1—Mo1—O4	109.24 (8)	N3—C23—H23A	108.5
O3—Mo1—O2	109.39 (8)	N3—C23—H23B	108.5
O4—Mo1—O2	109.01 (7)	H23A—C23—H23B	107.5
O4—Mo1—O3	109.70 (8)	C24—C23—N3	115.0 (2)
O5—Mo2—O6	109.75 (9)	C24—C23—H23A	108.5
O5—Mo2—O7	109.59 (8)	C24—C23—H23B	108.5
O5—Mo2—O8	109.20 (8)	C23—C24—H24A	109.5
O6—Mo2—O7	109.02 (8)	C23—C24—H24B	109.5
O6—Mo2—O8	109.83 (7)	C23—C24—H24C	109.5
O7—Mo2—O8	109.44 (7)	H24A—C24—H24B	109.5
H1WA—O1W—H1WB	107 (2)	H24A—C24—H24C	109.5
H2WA—O2W—H2WB	109 (2)	H24B—C24—H24C	109.5
H3WA—O3W—H3WB	107 (2)	N4—C25—H25A	108.5
H4WA—O4W—H4WB	108 (2)	N4—C25—H25B	108.5
C3—N1—C1	111.92 (18)	H25A—C25—H25B	107.5
C3—N1—C5	108.56 (17)	C26—C25—N4	114.9 (2)
C5—N1—C1	107.80 (16)	C26—C25—H25A	108.5
C7—N1—C1	108.30 (16)	C26—C25—H25B	108.5
C7—N1—C3	108.21 (17)	C25—C26—H26A	109.5
C7—N1—C5	112.10 (17)	C25—C26—H26B	109.5
N1—C1—H1A	108.3	C25—C26—H26C	109.5
N1—C1—H1B	108.3	H26A—C26—H26B	109.5
H1A—C1—H1B	107.4	H26A—C26—H26C	109.5
C2—C1—N1	115.85 (18)	H26B—C26—H26C	109.5
C2—C1—H1A	108.3	N4—C27—H27A	108.6
C2—C1—H1B	108.3	N4—C27—H27B	108.6
C1—C2—H2A	109.5	H27A—C27—H27B	107.6
C1—C2—H2B	109.5	C28—C27—N4	114.7 (3)
C1—C2—H2C	109.5	C28—C27—H27A	108.6
H2A—C2—H2B	109.5	C28—C27—H27B	108.6
H2A—C2—H2C	109.5	C27—C28—H28A	109.5
H2B—C2—H2C	109.5	C27—C28—H28B	109.5
N1—C3—H3A	108.6	C27—C28—H28C	109.5
N1—C3—H3B	108.6	H28A—C28—H28B	109.5
N1—C3—C4	114.8 (2)	H28A—C28—H28C	109.5
H3A—C3—H3B	107.6	H28B—C28—H28C	109.5
C4—C3—H3A	108.6	N4—C29—H29A	108.3
C4—C3—H3B	108.6	N4—C29—H29B	108.3
C3—C4—H4A	109.5	H29A—C29—H29B	107.4
C3—C4—H4B	109.5	C30—C29—N4	115.7 (2)
C3—C4—H4C	109.5	C30—C29—H29A	108.3
H4A—C4—H4B	109.5	C30—C29—H29B	108.3
H4A—C4—H4C	109.5	C29—C30—H30A	109.5
H4B—C4—H4C	109.5	C29—C30—H30B	109.5
N1—C5—H5A	108.5	C29—C30—H30C	109.5
N1—C5—H5B	108.5	H30A—C30—H30B	109.5
H5A—C5—H5B	107.5	H30A—C30—H30C	109.5
C6—C5—N1	115.1 (2)	H30B—C30—H30C	109.5
C6—C5—H5A	108.5	N4—C31—H31A	108.6
C6—C5—H5B	108.5	N4—C31—H31B	108.6
C5—C6—H6A	109.5	N4—C31—C32	114.8 (2)
C5—C6—H6B	109.5	H31A—C31—H31B	107.5
C5—C6—H6C	109.5	C32—C31—H31A	108.6
H6A—C6—H6B	109.5	C32—C31—H31B	108.6
H6A—C6—H6C	109.5	C31—C32—H32A	109.5
H6B—C6—H6C	109.5	C31—C32—H32B	109.5
N1—C7—H7A	108.5	C31—C32—H32C	109.5
N1—C7—H7B	108.5	H32A—C32—H32B	109.5
H7A—C7—H7B	107.5	H32A—C32—H32C	109.5
C8—C7—N1	114.91 (19)	H32B—C32—H32C	109.5
C8—C7—H7A	108.5	C17B—N3B—C19B	111 (2)
C8—C7—H7B	108.5	C21B—N3B—C17B	115 (2)
C7—C8—H8A	109.5	C21B—N3B—C19B	101 (2)
C7—C8—H8B	109.5	C21B—N3B—C23B	115 (2)
C7—C8—H8C	109.5	C23B—N3B—C17B	105 (2)
H8A—C8—H8B	109.5	C23B—N3B—C19B	110 (2)
H8A—C8—H8C	109.5	N3B—C17B—H17C	108.4
H8B—C8—H8C	109.5	N3B—C17B—H17D	108.4
C11—N2—C9	111.96 (17)	H17C—C17B—H17D	107.5
C11—N2—C13	108.74 (16)	C18B—C17B—N3B	115.5 (15)
C11—N2—C15	107.81 (16)	C18B—C17B—H17C	108.4
C13—N2—C9	107.91 (15)	C18B—C17B—H17D	108.4
C15—N2—C9	108.48 (15)	C17B—C18B—H18D	109.5
C15—N2—C13	111.99 (17)	C17B—C18B—H18E	109.5
N2—C9—H9A	108.4	C17B—C18B—H18F	109.5
N2—C9—H9B	108.4	H18D—C18B—H18E	109.5
H9A—C9—H9B	107.5	H18D—C18B—H18F	109.5
C10—C9—N2	115.39 (17)	H18E—C18B—H18F	109.5
C10—C9—H9A	108.4	N3B—C19B—H19C	108.4
C10—C9—H9B	108.4	N3B—C19B—H19D	108.4
C9—C10—H10A	109.5	H19C—C19B—H19D	107.4
C9—C10—H10B	109.5	C20B—C19B—N3B	115.6 (15)
C9—C10—H10C	109.5	C20B—C19B—H19C	108.4
H10A—C10—H10B	109.5	C20B—C19B—H19D	108.4
H10A—C10—H10C	109.5	C19B—C20B—H20D	109.5
H10B—C10—H10C	109.5	C19B—C20B—H20E	109.5
N2—C11—H11A	108.5	C19B—C20B—H20F	109.5
N2—C11—H11B	108.5	H20D—C20B—H20E	109.5
H11A—C11—H11B	107.5	H20D—C20B—H20F	109.5
C12—C11—N2	115.09 (18)	H20E—C20B—H20F	109.5
C12—C11—H11A	108.5	N3B—C21B—H21C	108.3
C12—C11—H11B	108.5	N3B—C21B—H21D	108.3
C11—C12—H12A	109.5	H21C—C21B—H21D	107.4
C11—C12—H12B	109.5	C22B—C21B—N3B	116.1 (15)
C11—C12—H12C	109.5	C22B—C21B—H21C	108.3
H12A—C12—H12B	109.5	C22B—C21B—H21D	108.3
H12A—C12—H12C	109.5	C21B—C22B—H22D	109.5
H12B—C12—H12C	109.5	C21B—C22B—H22E	109.5
N2—C13—H13A	108.6	C21B—C22B—H22F	109.5
N2—C13—H13B	108.6	H22D—C22B—H22E	109.5
H13A—C13—H13B	107.6	H22D—C22B—H22F	109.5
C14—C13—N2	114.71 (18)	H22E—C22B—H22F	109.5
C14—C13—H13A	108.6	N3B—C23B—H23C	108.3
C14—C13—H13B	108.6	N3B—C23B—H23D	108.3
C13—C14—H14A	109.5	H23C—C23B—H23D	107.4
C13—C14—H14B	109.5	C24B—C23B—N3B	115.9 (15)
C13—C14—H14C	109.5	C24B—C23B—H23C	108.3
H14A—C14—H14B	109.5	C24B—C23B—H23D	108.3
H14A—C14—H14C	109.5	C23B—C24B—H24D	109.5
H14B—C14—H14C	109.5	C23B—C24B—H24E	109.5
N2—C15—H15A	108.5	C23B—C24B—H24F	109.5
N2—C15—H15B	108.5	H24D—C24B—H24E	109.5
N2—C15—C16	115.10 (18)	H24D—C24B—H24F	109.5
H15A—C15—H15B	107.5	H24E—C24B—H24F	109.5
C16—C15—H15A	108.5	C25B—N4B—C27B	102.7 (15)
C16—C15—H15B	108.5	C25B—N4B—C29B	115.6 (14)
C15—C16—H16A	109.5	C25B—N4B—C31B	111.1 (14)
C15—C16—H16B	109.5	C29B—N4B—C27B	111.2 (14)
C15—C16—H16C	109.5	C31B—N4B—C27B	109.7 (14)
H16A—C16—H16B	109.5	C31B—N4B—C29B	106.6 (15)
H16A—C16—H16C	109.5	N4B—C25B—H25C	108.5
H16B—C16—H16C	109.5	N4B—C25B—H25D	108.5
C19—N3—C17	111.3 (2)	H25C—C25B—H25D	107.5
C19—N3—C21	105.7 (2)	C26B—C25B—N4B	115.0 (12)
C19—N3—C23	111.71 (19)	C26B—C25B—H25C	108.5
C21—N3—C17	111.36 (19)	C26B—C25B—H25D	108.5
C23—N3—C17	106.45 (19)	C25B—C26B—H26D	109.5
C23—N3—C21	110.5 (2)	C25B—C26B—H26E	109.5
C27—N4—C25	105.3 (2)	C25B—C26B—H26F	109.5
C29—N4—C25	110.6 (2)	H26D—C26B—H26E	109.5
C29—N4—C27	111.5 (2)	H26D—C26B—H26F	109.5
C29—N4—C31	106.5 (2)	H26E—C26B—H26F	109.5
C31—N4—C25	111.4 (2)	N4B—C27B—H27C	108.8
C31—N4—C27	111.5 (2)	N4B—C27B—H27D	108.8
N3—C17—H17A	108.4	H27C—C27B—H27D	107.7
N3—C17—H17B	108.4	C28B—C27B—N4B	113.9 (16)
H17A—C17—H17B	107.4	C28B—C27B—H27C	108.8
C18—C17—N3	115.7 (2)	C28B—C27B—H27D	108.8
C18—C17—H17A	108.4	C27B—C28B—H28D	109.5
C18—C17—H17B	108.4	C27B—C28B—H28E	109.5
C17—C18—H18A	109.5	C27B—C28B—H28F	109.5
C17—C18—H18B	109.5	H28D—C28B—H28E	109.5
C17—C18—H18C	109.5	H28D—C28B—H28F	109.5
H18A—C18—H18B	109.5	H28E—C28B—H28F	109.5
H18A—C18—H18C	109.5	N4B—C29B—H29C	108.2
H18B—C18—H18C	109.5	N4B—C29B—H29D	108.2
N3—C19—H19A	108.5	H29C—C29B—H29D	107.4
N3—C19—H19B	108.5	C30B—C29B—N4B	116.2 (13)
N3—C19—C20	115.1 (2)	C30B—C29B—H29C	108.2
H19A—C19—H19B	107.5	C30B—C29B—H29D	108.2
C20—C19—H19A	108.5	C29B—C30B—H30D	109.5
C20—C19—H19B	108.5	C29B—C30B—H30E	109.5
C19—C20—H20A	109.5	C29B—C30B—H30F	109.5
C19—C20—H20B	109.5	H30D—C30B—H30E	109.5
C19—C20—H20C	109.5	H30D—C30B—H30F	109.5
H20A—C20—H20B	109.5	H30E—C30B—H30F	109.5
H20A—C20—H20C	109.5	N4B—C31B—H31C	108.3
H20B—C20—H20C	109.5	N4B—C31B—H31D	108.3
N3—C21—H21A	108.6	H31C—C31B—H31D	107.4
N3—C21—H21B	108.6	C32B—C31B—N4B	116.1 (12)
H21A—C21—H21B	107.6	C32B—C31B—H31C	108.3
C22—C21—N3	114.8 (2)	C32B—C31B—H31D	108.3
C22—C21—H21A	108.6	C31B—C32B—H32D	109.5
C22—C21—H21B	108.6	C31B—C32B—H32E	109.5
C21—C22—H22A	109.5	C31B—C32B—H32F	109.5
C21—C22—H22B	109.5	H32D—C32B—H32E	109.5
C21—C22—H22C	109.5	H32D—C32B—H32F	109.5
H22A—C22—H22B	109.5	H32E—C32B—H32F	109.5
			
C1—N1—C3—C4	−55.1 (3)	C25—N4—C27—C28	−178.8 (3)
C1—N1—C5—C6	−64.4 (3)	C25—N4—C29—C30	58.6 (4)
C1—N1—C7—C8	172.7 (2)	C25—N4—C31—C32	−58.4 (4)
C3—N1—C1—C2	−57.0 (3)	C27—N4—C25—C26	−177.6 (3)
C3—N1—C5—C6	174.2 (2)	C27—N4—C29—C30	−58.2 (4)
C3—N1—C7—C8	−65.8 (3)	C27—N4—C31—C32	59.0 (4)
C5—N1—C1—C2	−176.3 (2)	C29—N4—C25—C26	61.8 (3)
C5—N1—C3—C4	63.8 (3)	C29—N4—C27—C28	−58.8 (4)
C5—N1—C7—C8	53.9 (3)	C29—N4—C31—C32	−179.1 (3)
C7—N1—C1—C2	62.2 (3)	C31—N4—C25—C26	−56.5 (3)
C7—N1—C3—C4	−174.3 (2)	C31—N4—C27—C28	60.2 (4)
C7—N1—C5—C6	54.7 (3)	C31—N4—C29—C30	179.9 (3)
C9—N2—C11—C12	−56.9 (3)	C17B—N3B—C19B—C20B	−63 (4)
C9—N2—C13—C14	−62.6 (3)	C17B—N3B—C21B—C22B	57 (4)
C9—N2—C15—C16	172.0 (2)	C17B—N3B—C23B—C24B	−179 (4)
C11—N2—C9—C10	−55.6 (2)	C19B—N3B—C17B—C18B	−71 (5)
C11—N2—C13—C14	175.7 (2)	C19B—N3B—C21B—C22B	177 (4)
C11—N2—C15—C16	−66.6 (3)	C19B—N3B—C23B—C24B	62 (5)
C13—N2—C9—C10	−175.20 (19)	C21B—N3B—C17B—C18B	43 (5)
C13—N2—C11—C12	62.3 (3)	C21B—N3B—C19B—C20B	175 (4)
C13—N2—C15—C16	53.0 (3)	C21B—N3B—C23B—C24B	−52 (5)
C15—N2—C9—C10	63.3 (2)	C23B—N3B—C17B—C18B	170 (4)
C15—N2—C11—C12	−176.1 (2)	C23B—N3B—C19B—C20B	53 (4)
C15—N2—C13—C14	56.7 (3)	C23B—N3B—C21B—C22B	−65 (4)
C17—N3—C19—C20	63.2 (3)	C25B—N4B—C27B—C28B	−176 (2)
C17—N3—C21—C22	−59.4 (3)	C25B—N4B—C29B—C30B	−57 (3)
C17—N3—C23—C24	−178.9 (3)	C25B—N4B—C31B—C32B	55 (2)
C19—N3—C17—C18	59.1 (3)	C27B—N4B—C25B—C26B	−178 (2)
C19—N3—C21—C22	179.6 (2)	C27B—N4B—C29B—C30B	59 (2)
C19—N3—C23—C24	−57.2 (3)	C27B—N4B—C31B—C32B	−58 (2)
C21—N3—C17—C18	−58.5 (3)	C29B—N4B—C25B—C26B	−57 (3)
C21—N3—C19—C20	−175.8 (2)	C29B—N4B—C27B—C28B	60 (3)
C21—N3—C23—C24	60.1 (3)	C29B—N4B—C31B—C32B	−178.7 (19)
C23—N3—C17—C18	−178.9 (3)	C31B—N4B—C25B—C26B	65 (2)
C23—N3—C19—C20	−55.6 (3)	C31B—N4B—C27B—C28B	−58 (3)
C23—N3—C21—C22	58.7 (3)	C31B—N4B—C29B—C30B	179 (2)

### Bis(tetraethylammonium) molybdate dihydrate (200k) Hydrogen-bond geometry (Å, º)

**Table:**

*D*—H···*A*	*D*—H	H···*A*	*D*···*A*	*D*—H···*A*
C1—H1*A*···O2*W*	0.99	2.46	3.425 (3)	166
C1—H1*B*···O3	0.99	2.42	3.346 (3)	156
C5—H5*A*···O5	0.99	2.46	3.399 (3)	158
C5—H5*B*···O4*W*	0.99	2.52	3.399 (3)	148
C7—H7*A*···O4^i^	0.99	2.52	3.469 (3)	160
C9—H9*A*···O1*W*^ii^	0.99	2.51	3.443 (3)	156
C9—H9*B*···O7	0.99	2.40	3.310 (3)	153
C10—H10*A*···O6^i^	0.98	2.46	3.432 (3)	172
C11—H11*B*···O2^iii^	0.99	2.56	3.500 (3)	158
C13—H13*A*···O1^ii^	0.99	2.54	3.341 (3)	138
C13—H13*B*···O3*W*^ii^	0.99	2.58	3.461 (3)	148
C15—H15*B*···O6^i^	0.99	2.57	3.410 (3)	143
C19—H19*B*···O1^iv^	0.99	2.56	3.258 (3)	128
C21—H21*B*···O1*W*^iv^	0.99	2.58	3.555 (3)	167
C23—H23*B*···O8	0.99	2.56	3.531 (3)	167
C25—H25*B*···O4*W*^v^	0.99	2.45	3.430 (3)	172
C27—H27*B*···O7	0.99	2.48	3.173 (3)	127
C29—H29*B*···O3^vi^	0.99	2.40	3.362 (3)	163
C32—H32*A*···O3*W*^vii^	0.98	2.55	3.220 (4)	125

Symmetry codes: (i) *x*−1, *y*, *z*; (ii) *x*, *y*−1, *z*; (iii) *x*−1, *y*−1, *z*; (iv) −*x*+1, −*y*+1, −*z*+1; (v) *x*+1, *y*, *z*; (vi) −*x*+1, −*y*+1, −*z*; (vii) −*x*, −*y*+1, −*z*.
